# The Poincaré-Shannon Machine: Statistical Physics and Machine Learning Aspects of Information Cohomology

**DOI:** 10.3390/e21090881

**Published:** 2019-09-10

**Authors:** Pierre Baudot

**Affiliations:** 1 Inserm UNIS UMR1072—Université Aix-Marseille, 13015 Marseille, France; pierre.baudot@mediantechnologies.com; Tel.: +33-6-19-39-10-39; 2 Median Technologies, 06560 Valbonne, France

**Keywords:** algebraic topology, machine learning, information theory, statistical physics, deep neural networks, unsupervised learning, multivariate mutual information, statistical dependences, k-body interactions, synergy, clustering

## Abstract

Previous works established that entropy is characterized uniquely as the first cohomology class in a topos and described some of its applications to the unsupervised classification of gene expression modules or cell types. These studies raised important questions regarding the statistical meaning of the resulting cohomology of information and its interpretation or consequences with respect to usual data analysis and statistical physics. This paper aims to present the computational methods of information cohomology and to propose its interpretations in terms of statistical physics and machine learning. In order to further underline the cohomological nature of information functions and chain rules, the computation of the cohomology in low degrees is detailed to show more directly that the *k* multivariate mutual information ($I_{k}$) are (k−1)-coboundaries. The (k−1)-cocycles condition corresponds to $I_{k}=0$, which generalizes statistical independence to arbitrary degree *k*. Hence, the cohomology can be interpreted as quantifying the statistical dependences and the obstruction to factorization. I develop the computationally tractable subcase of simplicial information cohomology represented by entropy $H_{k}$ and information $I_{k}$ landscapes and their respective paths, allowing investigation of Shannon’s information in the multivariate case without the assumptions of independence or of identically distributed variables. I give an interpretation of this cohomology in terms of phase transitions in a model of *k*-body interactions, holding both for statistical physics without mean field approximations and for data points. The $I_{1}$ components define a self-internal energy functional $U_{k}$ and ${\left(-1\right)}^{k}I_{k,k\geq 2}$ components define the contribution to a free energy functional $G_{k}$ (the total correlation) of the *k*-body interactions. A basic mean field model is developed and computed on genetic data reproducing usual free energy landscapes with phase transition, sustaining the analogy of clustering with condensation. The set of information paths in simplicial structures is in bijection with the symmetric group and random processes, providing a trivial topological expression of the second law of thermodynamics. The local minima of free energy, related to conditional information negativity and conditional independence, characterize a minimum free energy complex. This complex formalizes the minimum free-energy principle in topology, provides a definition of a complex system and characterizes a multiplicity of local minima that quantifies the diversity observed in biology. I give an interpretation of this complex in terms of unsupervised deep learning where the neural network architecture is given by the chain complex and conclude by discussing future supervised applications.

1 Introduction3   1.1 Observable Physics of Information3   1.2 Statistical Interpretation: Hierarchical Independences and Dependences Structures3   1.3 Statistical Physics Interpretation: *K*-Body Interacting Systems4   1.4 Machine Learning Interpretation: Topological Deep Learning52 Information Cohomology6   2.1 A Long March through Information Topology6   2.2 Information Functions (Definitions)7   2.3 Information Structures and Coboundaries10      2.3.1 First Degree (k=1)12      2.3.2 Second Degree (k=2)12      2.3.3 Third Degree (k=3)12      2.3.4 Higher Degrees133 Simplicial Information Cohomology13   3.1 Simplicial Substructures of Information13   3.2 Topological Self and Free Energy of *K*-Body Interacting System-Poincaré-Shannon Machine14   3.3 *k*-Entropy and *k*-Information Landscapes18   3.4 Information Paths and Minimum Free Energy Complex19      3.4.1 Information Paths (Definition)19      3.4.2 Derivatives, Inequalities and Conditional Mutual-Information Negativity20      3.4.3 Information Paths Are Random Processes: Topological Second Law of Thermodynamics and Entropy Rate21      3.4.4 Local Minima and Critical Dimension23      3.4.5 Sum over Paths and Mean Information Path24      3.4.6 Minimum Free Energy Complex254 Discussion27   4.1 Statistical Physics27      4.1.1 Statistical Physics without Statistical Limit? Complexity through Finite Dimensional Non-Extensivity27      4.1.2 Naive Estimations Let the Data Speak28      4.1.3 Discrete Informational Analog of Renormalization Methods: No Mean-Field Assumptions Let the Objects Differentiate29      4.1.4 Combinatorial, Infinite, Continuous and Quantum Generalizations29   4.2 Data Science29      4.2.1 Topological Data Analysis29      4.2.2 Unsupervised and Supervised Deep Homological Learning30      4.2.3 Epigenetic Topological Learning—Biological Diversity315 Conclusions32References33

*“Now what is science? ...it is before all a classification, a manner of bringing together facts which appearances separate, though they are bound together by some natural and hidden kinship. Science, in other words, is a system of relations. ...it is in relations alone that objectivity must be sought. ...it is relations alone which can be regarded as objective. External objects... are really objects and not fleeting and fugitive appearances, because they are not only groups of sensations, but groups cemented by a constant bond. It is this bond, and this bond alone, which is the object in itself, and this bond is a relation.”*

H. Poincaré

## 1. Introduction

The present paper aims to provide a comprehensive introduction and interpretation in terms of statistics, statistical physics and machine learning of the information cohomology theory developed in References [1,2]. It presents the computational aspects of the application of information cohomology to data presented in Reference [3] and in the associated paper [2], which consists of an unsupervised classification of cell types or gene modules and provides a generic model for epigenetic co-regulation and differentiation.

### 1.1. Observable Physics of Information

In its application to empirically measured data, information cohomology is at the cross-roads of both data analysis and statistical physics and this article aims to give some keys to its interpretation within those two fields, which could be quoted as “passing the information between disciplines” in reference to Mezard’s review [4]. Just as topos have been used as a communication bridge allowing the translation of theorems between different domains and therefore to unify mathematical theories [5], information cohomology can help in further unraveling some equivalences between different disciplines (e.g., statistical physics and machine learning) and shall play a foundational role in both as already proposed by Doering and Isham considering only probability structures [6,7]. In doing so, information theory goes one step toward a general mathematical and physical theory of communication (or of measure and observation), where information conservation is an isomorphism. In other terms, following the paths of topology, this paper pursues the work that started with the studies of Brillouin [8], Jaynes [9,10], Landauer [11], Penrose [12], Wheeler [13], and Bennett [14], of an informational theory of physics that would be furthermore restricted to a theory of data: an austere empirical theory [15] with a minimum set of priors or axioms, a physics that “let the data speak”. It is the axiom of observability (“*concepts which correspond to no conceivable observation should be eliminated from physics*” [16], p. 264) which imposes that statistical physics and data sciences shall not be dissociated but unified in their foundations. Thermodynamics established that free energy is the energy that can be effectively used, whereas entropy was also called “lost heat”. The same holds generically on whatever data with mutual information and total correlations, some kind of relative or shared information: mutual information is the information that can be effectively used for pattern detection and classification. It hence appears that knowledge is a form of energy [17] and the results suggest that there are important resources of such information-energy in the *k*-body dependences even beyond pairwise interactions.

### 1.2. Statistical Interpretation: Hierarchical Independences and Dependences Structures

First, in order to provide a mathematical interpretation of information cohomology, I give a brief bibliographical overview of its multiple and diverse origins coming notably from the theory of motive and recall the open conjecture on the higher classes. In References [1,2], entropy was characterized uniquely as the first cohomology class in a topos theory and this result could be extended to quantum information, Kullback-Leibler divergence and cross-entropy (Proposition 4 [1]) and have been further developed and extended by Vigneaux [18,19] to Tsallis entropies and differential entropy following some preliminary results of Marcolli and Thorngren [20]. Here, we show directly by computing the cohomology in the low degrees that multivariate *k*-mutual information, denoted $I_{k}$, are (k−1)-coboundaries. Recalling a result of the associated paper (Theorem 2 [2]), establishing that *n* random variables are independent if and only if all the subsets of *k*-mutual information vanish (k≥2), allows one to conclude that the (k−1)-cocycles condition corresponds to $I_{k}=0$, which generalizes statistical independence to an arbitrary degree *k*. The interpretation and meaning of information cohomology is hence the quantification of the statistical dependences and of the obstruction to factorization—a result confirmed by the work of Mainiero, with a different approach [21]. Notably, the introduction of a symmetric action of conditioning, following Gerstenhaber and Schack [22], gives a 1-cocycle condition that characterizes the famous information pseudo-metric discovered by Shannon, Rajski, Zurek, Crutchfield and Bennett [23,24,25,26,27,28] and that can be generalized to the *k*-multivariate case by new symmetric non-negative information functions, the pseudo *k*-volumes $V_{k}=H_{k}-I_{k}$.

### 1.3. Statistical Physics Interpretation: *K*-Body Interacting Systems

Second, to provide an interpretation of information cohomology in terms of statistical physics, this paper settles information structures in the context of a generic *k*-body interacting system. One can remark that the setting of information cohomology is equivalent to the Potts models that generalizes the spin models to arbitrary multivalued variables (see Reference [29] for review) and considers all possible *k*-ary statistical interactions in a similar way as the multispin interaction models do (*k*-spin interaction models that generalize pairwise and nearest-neighbor models, see Reference [30] and reference therein). I describe the computational and combinatorial restrictions from the lattice of partitions to the simplicial sub-lattice that allows one to compute in practice the information cohomology on data as in References [2,3] and that defines entropy and information landscapes and their respective paths that provide the discrete informational analog of path integrals. The combinatorics of possible interactions that are computed by the information landscapes is equivalent to the computational *“exponential wall”* encountered in many particle studies, notably density functional theory (DFT), as exposed by Kohn [31]. The $I_{1}$ component defines a self-internal energy functional $U_{k}$ and ${\left(-1\right)}^{k}I_{k,k\geq 2}$ components define the contribution to a total free-energy functional $G_{k}$ of the *k*-body interactions (i.e., the total correlation). The total free energy is a Kullback-Leibler divergence and a special case (symmetric in the interacting body) of the free energy introduced by Baez and Pollard [32] (see also the appendix of the associated paper [2]). These definitions allow the recovery of usual equilibrium or semi-classical expressions in special cases. The set of all first critical points of information paths—a conditional-independence condition—gives a construction of the minimum free energy complex. Mutual information negativity, also called synergy [33]—a phenomenon known to provide the signature of frustrated states in glasses since the work of Matsuda [34]—is related here in the context of the more general conditional mutual information to a kind of (discrete) first-order transition, analogous to smooth phase transition in small systems [35], yet seen topologically as the critical points of a simplicial complex.

To further settle the thermodynamical interpretation of information cohomology, it is relevant to wonder what the cohomological expression of the first and second principles of thermodynamics could be. The set of information paths in simplicial structures is in bijection with the symmetric group and random processes, providing a trivial topological expression of the second law of thermodynamic as a consequence of entropy convexity, improving the theorem of Cover that needed to assume Markov conditions [36]. Thanks to the theorem of Noether [37], the first principle is expressed in terms of continuous symmetries and her theorem has been restated in more modern homological terms for finite elements by Mansfield [38] and for Markov chains by Baez and Fong [39]. Hence, the expression of the first principle in information cohomology, let as a conjecture here, should take the form of a Noether theorem for random discrete processes—that is, for the entire symmetric group $S_{n}$, a question already asked by Neuenschwander [40].

As presented by Kadanov [41] or in References [42,43], close to phase transition (notably in three dimensions), the failure of the mean field theories introduced by Van der Waals, Maxwell and Landau led to the development of renormalization group methods. Renormalization methods intrinsically rely on asymptotic unrealistic assumptions such as an infinite number of particles [41,43] and neglect infinite quantities, which has raised fundamental criticism [44,45]. In order to compute an analog of a mean field model like van der Waals interactions [46,47,48], we define and compute the mean information paths that correspond to the homogeneous case of identically distributed random variables. On a dataset of expected interacting genes, the mean information path reproduces the usual behavior of the free energy in the condensed phase (i.e., with a critical point), while for genes that are less expected to interact, the path exhibits a monotonic decrease without a non-trivial minimum which corresponds to the usual free-energy potential in the uncondensed disordered phase for which the *n*-body interactions are negligible. However, compared with the non-averaged original information paths as presented in References [2,3], it is clear that this analog mean-field approach erases the multiplicity of critical points and the diversity or richness of the complexes.

Hence, with respect to statistical physics, the main novelty brought by the information cohomology approach is the introduction of purely finite and discrete methods that can account for transition phenomena in heterogeneous systems without mean field assumptions and of new measures of correlations (i.e., multivariate mutual information that generalizes the usual correlation coefficients to non-linear relations) [2,3]. Among the work left for further studies in this preliminary line, I conjecture that the infinite dimensional continuous extension of the information cohomology formalism should be equivalent to renormalization methods, while I underline that, in practice, there is no need of these physically unrealistic assumptions.

### 1.4. Machine Learning Interpretation: Topological Deep Learning

The interpretation of the information cohomology in terms of machine learning and data analysis first relies on the definition of random variables as partition and of information structures and complexes on the lattice of partition [1]. Partitions are equivalent to equivalence classes (see Ellerman [49,50] for review) and hence the complex of random variables spans all possible equivalence classes of the data point. Therefore, the information cochain complex deserves the function of a universal classifier. Moreover, these information structures defined on the whole lattice of partitions encompass all possible statistical dependences and relations, since by definition it considers all possible equivalent classes on a probability space and hence fully answers to the problem raised by James and Crutchfield [51], who remarked on some partitions that are not distinguished by the $I_{k}$ simplicial structure. The combinatorics of these structures forbid any computation in practice, until quantum computers become available and the severe restriction to the simplicial case of the cohomology with complexity $O(2^{n}))$ implies that not all statistical dependencies can be estimated, as shown by James and Crutchfield [51]. The interpretation makes two phenomena coincide (i.e., condensation in statistical physics and clustering of data points in data science), which are signed by information and conditional information negativity as studied in the companion paper [2]. This generalizes the idea and results obtained on networks by the team of Grassberger [52].

On the side of applied algebraic topology, the identification of the topological structures of a dataset has motivated important research following the development of persistent homology [53,54,55]. Combining statistical and topological structures in a single framework remains an active challenge of data analysis that has already yielded some interesting results [56,57]. Some recent works have proposed information theoretical approaches grounded on homology, defining persistent entropy [58,59], graph topological entropy [60], spectral entropy [61] or multilevel integration entropies [62]. The present work is formally different and arose independent of persistence and provides a cohomology intrinsically based on probability for which the invariants are arguably the most important features of statistics and free of any metric assumption. The interpretation in terms of deep neural networks is also straightforward. It is preliminarily developed and applied here and in Reference [2] for the unsupervised case, while the supervised subcase is only briefly discussed here and left for further work. See Reference [63] for a presentation and preliminary applications to the digit images database of the Mixed National Institute of Standards and Technology (MNIST). The informational approach of topological data analysis provides a direct probabilistic and statistical analysis of the structure of a dataset which allows the gap with neural network analysis to be bridged and may be a step toward their formalization in mathematics and the characterization of the network architecture necessary for a given dataset. The original work based on spin networks by Hopfield [64] formalized fully recurrent networks as *n* binary random variables (N=2). Ackley, Hinton and Sejnowski [65] followed up by imposing the Markov Field condition, allowing the introduction of conditional independence to handle network structures with hidden layers and hidden nodes. The result—the Boltzmann or Helmholtz machine [66]—relies on the maximum entropy or free-energy minimization principle and originally on minimizing the relative entropy between the network and environmental states [65]. Indeed, Reference [67] and references therein (notably see the whole opus of Marcolli with application to linguistics [68]) provides a review of the relevance of homology to artificial or natural cognition. Considering neurons as binary random variables or more generally *N*-ary variables (corresponding to a rate coding hypothesis) in the present context provides a homologically constrained approach of those neural networks, where the first input layer is represented by the marginal (single variable, degree 1 component) while hidden layers are associated to higher degrees. In a very naive sense, higher cohomological degrees distinguish higher-order patterns (or higher-dimensional patterns in the simplicial case), just as receptive fields of convolutional neural networks recognize higher-order features when going to higher depth-rank of neural layers as described in David Marr’s original sketch [69] and now implemented efficiently in deep network structures. Notably, the notion of geodesic used in machine learning is replaced by the homotopical notion of path. On the data analysis side, it provides a new algorithm and tools for topological data analysis allowing one to rank and detect clusters and functional modules, and to make dimensionality reduction; indeed, all these classical tasks in data analysis have a direct homological meaning. I propose to call the data analysis method presented here the Poincaré-Shannon machine, since it implements simplicial homology (see Poincaré’s Analysis Situs [70]) and information theory in a single framework (see Shannon’s theory of communication [71]), applied effectively to empirical data.

## 2. Information Cohomology

This section provides a short bibliographical note on the inscription of information and probability theory within homological theories Section 2.1. We also recall the definition of information functions Section 2.2 and provide a short description of information cohomology computed in the low degrees Section 2.3, such that the interpretation of entropy and mutual information within Hochschild cohomology appears straightforward and clear. There are no new results in this section but I hope to provide a more simple and helpful presentation for some researchers outside the field of topology of what can be found in References [1,18,19] that should be considered for more precise and detailed exposition.

### 2.1. A Long March through Information Topology

From the mathematical point of view, a motivation of information topology is to capture the ambiguity theory of Galois, which is the essence of group theory or discrete symmetries (see André’s reviews [72,73]) and Shannon’s information uncertainty theory in a common framework—a path already paved by some results on information inequalities (see Yeung’s results [74]) and in algebraic geometry. In the work of Cathelineau, [75], entropy first appeared in the computation of the degree-one homology of the discrete group SL(2,C) with coefficients in the adjoint action by choosing a pertinent definition of the derivative of the Bloch–Wigner dilogarithm. It could be shown that the functional equation with five terms of the dilogarithm implies the functional equation of entropy with four terms. Kontsevitch [76] discovered that a finite truncated version of the logarithm appearing in cyclotomic studies also satisfied the functional equation of entropy, suggesting a higher-degree generalization of information, analog to polylogarithm and hence showing that the functional equation of entropy holds in *p* and 0 field characteristics. Elbaz-Vincent and Gangl used algebraic means to construct this information generalization which holds over finite fields [77], where information functions appear as derivations [78]. After entropy appeared in tropical and idempotent semi-ring analysis in the study of the extension of Witt semiring to the characteristic 1 limit [79], Marcolli and Thorngren developed the thermodynamic semiring, an entropy operad that could be constructed as a deformation of the tropical semiring [20]. Introducing Rota–Baxter algebras, it allowed the derivation of a renormalization procedure [80]. In defining the category of finite probability and using Fadeev axiomatization, Baez, Fritz and Leinster could show that the only family of functions that has the functorial property is Shannon information loss [81,82]. Basing his approach on information and Koszul geometry, Boyom developed a more geometrical view of statistical models that notably considers foliations in place of the random variables [83]. Introducing a deformation theoretic framework and chain complex of random variables, Drumond-Cole, Park and Terilla [84,85,86] constructed a homotopy probability theory for which the cumulants coincide with the morphisms of the homotopy algebras. The probabilistic framework used here was introduced in Reference [1] and generalized to Tsallis entropies by Vigneaux [18,19]. The diversity of the formalisms employed in these independent but convergent approaches is astonishing. So, as to the question “what is information topology?”, it is only possible to answer that it is under development at the moment. The results of Catelineau, Elbaz-Vincent and Gangl inscribed information into the theory of motives, which according to Beilison’s program is a mixed Hodge-Tate cohomology [87]. All along the development of the application to data, following the cohomology developed by References [1,18] on an explicit probabilistic basis, we aimed to preserve such a structure and unravel its expression in information theoretic terms. Moreover, following Aomoto’s results [88,89], the actual conjecture [1] is that the higher classes of information cohomology should be some kind of polylogarithmic *k*-form (*k*-differential volumes that are symmetric and additive and that correspond to the cocycle conditions for the cohomology of Lie groups [88]). The following developments suggest that these higher information groups should be the families of functions satisfying the functional equations of *k*-independence $I_{k}=0$− a rather vague but intuitive view that can be tested in special cases.

### 2.2. Information Functions (Definitions)

The information functions used in Reference [1] and the present study were originally defined by Shannon [71] and Kullback [90] and further generalized and developed by Hu Kuo Ting [91] and Yeung [92] (see also McGill [93]). These functions include entropy, denoted $H_{1}=H\left(X;P\right)$; joint entropy, denoted $H_{k}=H\left(X_{1},\ldots ,X_{k};P\right)$; mutual information, denoted $I_{2}=I\left(X_{1};X_{2};P\right)$; multivariate *k*-mutual information, denoted $I_{k}=I\left(X_{1};\ldots ;X_{k};P\right)$; and the conditional entropy and mutual information, denoted $Y.H_{k}=H\left(X_{1},\ldots ,X_{k}|Y;P\right)$ and $Y.I_{k}=I\left(X_{1};\ldots ;X_{k}|Y;P\right)$. The classical expression of these functions is the following (using k=−1/ln2, the usual bit unit):

- The Shannon-Gibbs entropy of a single variable $X_{j}$ is defined by [71]:
  (1) $$H_{1}=H\left(X_{j};P_{X_{j}}\right)=k\sum_{x\in [N_{j}]}p\left(x\right)\ln p\left(x\right)=k\sum_{i=1}^{N_{j}}p_{i}\ln p_{i},$$
  where $\left[N_{j}\right]=\left\{1,\ldots ,N_{j}\right\}$ denotes the alphabet of $X_{j}$.
- The relative entropy or Kullback-Liebler divergence, which was also called “discrimination information” by Kullback [90], is defined for two probability mass functions p(x) and q(x) by:
  (2) $$\begin{array}{cc} D(p(x)||q(x)) & =D\left(X;p\left(x\right)||q\left(x\right)\right)=k\sum_{x\in X}p\left(x\right)\ln\frac{q(x)}{p(x)}\\ \; & =H(X;p(x),q(x))-H(X;p(x)), \end{array}$$
  where H(X;p(x),q(x)) is the cross-entropy and H(X;p(x)) the Shannon entropy. It hence generates minus entropy as a special case, taking the deterministic constant probability q(x)=1. With the convention k=−1/ln2, D(p(x)||q(x)) is always positive or null.
- The joint entropy is defined for any joint product of *k* random variables $\left(X_{1},\ldots ,X_{k}\right)$ and for a probability joint distribution $P_{\left(X_{1},\ldots ,X_{k}\right)}$ by [71]:
  (3) $$\begin{array}{cc} H_{k} & =H(X_{1},\ldots ,X_{k};P_{X_{1},\ldots ,X_{k}})\\ \; & =k\sum_{x_{1},\ldots ,x_{k}\in \left[N_{1}\times \ldots \times N_{k}\right]}^{N_{1}\times \ldots \times N_{k}}p\left(x_{1}\ldots x_{k}\right)\ln p\left(x_{1}\ldots x_{k}\right)\\ \; & =k\sum_{i,j,\ldots ,k}^{N_{1},\ldots ,N_{k}}p_{\underset{k\;\mathrm{indices}}{\underset{︸}{ij\ldots k}}}\ln p_{ij\ldots k}, \end{array}$$
  where $\left[N_{1}\times \ldots \times N_{k}\right]=\left\{1,\ldots ,N_{j}\times \ldots \times N_{k}\right\}$ denotes the alphabet of $\left(X_{1},\ldots ,X_{k}\right)$.
- The mutual information of two variables $X_{1},X_{2}$ is defined as [71]:
  (4) $$I\left(X_{1};X_{2};P_{X_{1},X_{2}}\right)=k\sum_{x_{1},x_{2}\in \left[N_{1}\times N_{2}\right]}^{N_{1}\times N_{2}}p\left(x_{1}.x_{2}\right)\ln\frac{p\left(x_{1}\right)p\left(x_{2}\right)}{p(x_{1}.x_{2})},$$
  and it can be generalized to *k*-mutual information (also called co-information) using the alternated sums given by Equation (17), as originally defined by McGill [93] and Hu Kuo Ting [91], giving:
  (5) $$I_{k}=I\left(X_{1};\ldots ;X_{k};P\right)=k\sum_{x_{1},\ldots ,x_{k}\in \left[N_{1}\times \ldots \times N_{k}\right]}^{N_{1}\times \ldots \times N_{k}}p\left(x_{1}\ldots \ldots x_{k}\right)\ln\frac{\prod_{I\subset [k];card(I)=i;i\;\mathrm{odd}}p_{I}}{\prod_{I\subset [k];card(I)=i;i\;\mathrm{even}}p_{I}}.$$
  For example, the 3-mutual information is the function:
  (6) $$I_{3}=k\sum_{x_{1},x_{2},x_{3}\in \left[N_{1}\times N_{2}\times N_{3}\right]}^{N_{1}\times N_{2}\times N_{3}}p\left(x_{1}.x_{2}.x_{3}\right)\ln\frac{p\left(x_{1}\right)p\left(x_{2}\right)p\left(x_{3}\right)p\left(x_{1}.x_{2}.x_{3}\right)}{p\left(x_{1}.x_{2}\right)p\left(x_{1}.x_{3}\right)p\left(x_{2}.x_{3}\right)}.$$
  For k≥3, $I_{k}$ can be negative [91].
- The total correlation introduced by Watanabe [94] called integration by Tononi and Edelman [95] or multi-information by Studený and Vejnarova [96] and Margolin and colleagues [97], which we denote $C_{k}\left(X_{1};\ldots X_{k};P\right)$, is defined by:
  (7) $$\begin{array}{cc} C_{k} & =C_{k}\left(X_{1};\ldots X_{k};P\right)=\sum_{i=1}^{k}H\left(X_{i}\right)-H\left(X_{1};\ldots X_{k}\right)=\sum_{i=2}^{k}{\left(-1\right)}^{i}\sum_{I\subset [n];card(I)=i}I_{i}\left(X_{I};P\right)\\ \; & =k\sum_{x_{1},\ldots ,x_{k}\in \left[N_{1}\times \ldots \times N_{k}\right]}^{N_{1}\times \ldots \times N_{k}}p\left(x_{1}\ldots .x_{k}\right)\ln\frac{p(x_{1}\ldots x_{k})}{p\left(x_{1}\right)\ldots p\left(x_{k}\right)}. \end{array}$$
  For two variables, the total correlation is equal to the mutual information ($C_{2}=I_{2}$). The total correlation has the favorable property of being a relative entropy 2 between marginal and joint-variable and hence of being always non-negative.
- The conditional entropy of $X_{1}$ knowing (or given) $X_{2}$ is defined as [71]:
  (8) $$\begin{array}{c} X_{2}.H_{1}=H\left(X_{1}|X_{2};P\right)=k\sum_{x_{1},x_{2}\in \left[N_{1}\times N_{2}\right]}^{N_{1}*N_{2}}p\left(x_{1}.x_{2}\right)\ln p_{x_{2}}\left(x_{1}\right)\\ =k\sum_{x_{2}\in X_{2}}^{N_{2}}p\left(x_{2}\right).\left(\sum_{x_{1}\in X_{1}}^{N_{1}}p_{x_{2}}x_{1}\ln p_{x_{2}}x_{1}\right). \end{array}$$
  Conditional joint-entropy, $X_{3}.H\left(X_{1},X_{2}\right)$ or $\left(X_{1},X_{2}\right).H\left(X_{3}\right)$, is defined analogously by replacing the marginal probabilities by the joint probabilities.
- The conditional mutual information of two variables $X_{1},X_{2}$ knowing a third $X_{3}$ is defined as [71]:
  (9) $$X_{3}.I_{2}=I\left(X_{1};X_{2}|X_{3};P\right)=k\sum_{x_{1},x_{2},x_{3}\in \left[N_{1}\times N_{2}\times N_{3}\right]}^{N_{1}\times N_{2}\times N_{3}}p\left(x_{1}.x_{2}.x_{3}\right)\ln\frac{p_{x_{3}}\left(x_{1}\right)p_{x_{3}}\left(x_{2}\right)}{p_{x_{3}}\left(x_{1},x_{2}\right)}.$$
  Conditional mutual information generates all the preceding information functions as subcases, as shown by Yeung [92]. We have the theorem: if $X_{3}=\Omega$, then it gives the mutual information; if $X_{2}=X_{1}$, it gives conditional entropy; and if both conditions are satisfied, it gives entropy. Notably, we have $I_{1}=H_{1}$.

We now give the few information equalities and inequalities that are of central use in the homological framework, in the information diagrams and for the estimation of the information from the data.

We have the chain rules (see Reference [36] for proofs):

(10) $$H\left(X_{1};X_{2};P\right)=H\left(X_{1};P\right)+X_{1}.H\left(X_{2};P\right)=H\left(X_{2};P\right)+X_{2}.H\left(X_{1};P\right),$$

(11) $$I\left(X_{1};X_{2};P\right)=H\left(X_{1};P\right)-X_{2}.H\left(X_{1};P\right)=H\left(X_{2};P\right)-X_{1}.H\left(X_{2};P\right),$$

which we can write more generally as (where the hat denotes the omission of the variable):

(12) $$H\left(X_{1};\ldots ;\hat{X_{i}};\ldots ;X_{k+1};P\right)=H\left(X_{1};\ldots ;X_{k+1};P\right)-\left(X_{1};\ldots ;\hat{X_{i}};\ldots ;X_{k+1}\right).H\left(X_{i};P\right),$$

that we can write in short $H_{k+1}-H_{k}=\left(X_{1},\ldots X_{k}\right).H\left(X_{k+1}\right)$

(13) $$I\left(X_{1};\ldots ;\hat{X_{i}};\ldots ;X_{k+1};P\right)=I\left(X_{1};\ldots ;X_{k+1};P\right)+X_{i}.I\left(X_{1};\ldots ;\hat{X_{i}};\ldots ;X_{k+1};P\right),$$

which we can write in short $I_{k-1}-I_{k}=X_{k}.I_{k-1}$, generating the chain rule (10) as a special case.

These two equations provide recurrence relationships that give an alternative formulation of the chain rules in terms of a chosen path on the lattice of information structures:

(14) $$H_{k}=H\left(X_{1},\ldots ,X_{k};P\right)=\sum_{i=1}^{k}\left(X_{1},\ldots ,X_{i-1}\right).H\left(X_{i};P\right),$$

where we assume $H\left(X_{1};P\right)=X_{0}.H\left(X_{1};P\right)$ and hence that $X_{0}$ is the greatest element $X_{0}=\Omega$.

(15) $$I_{k}=I\left(X_{1};\ldots ;X_{k};P\right)=I\left(X_{1}\right)-\sum_{i=2}^{k}X_{i}.I\left(X_{1};\ldots ;X_{i-1}\right).$$

We have the alternated sums or inclusion–exclusion rules [1,34,91]:

(16) $$H_{n}\left(X_{1},\ldots ,X_{n};P\right)=\sum_{i=1}^{n}{\left(-1\right)}^{i-1}\sum_{I\subset [n];card(I)=i}I_{i}\left(X_{I};P\right),$$

(17) $$I_{n}\left(X_{1};\ldots ;X_{n};P\right)=\sum_{i=1}^{n}{\left(-1\right)}^{i-1}\sum_{I\subset [n];card(I)=i}H_{i}\left(X_{I};P\right).$$

For example: $H_{3}\left(X_{1},X_{2},X_{3}\right)=I_{1}\left(X_{1}\right)+I_{1}\left(X_{2}\right)+I_{1}\left(X_{3}\right)-I_{2}\left(X_{1};X_{2}\right)-I_{2}\left(X_{1};X_{3}\right)-I_{2}\left(X_{2};X_{3}\right)+I_{3}\left(X_{1};X_{2};X_{3}\right)$.

The chain rule of mutual information goes together with the following inequalities discovered by Matsuda [34]. For all random variables $X_{1};\ldots ;X_{k}$ with associated joint probability distribution *P*, we have the theorem due to Matsuda [34]

- $X_{k}.I\left(X_{1};\ldots ;X_{k-1};P\right)\geq 0$ if and only if $I\left(X_{1};\ldots ;X_{k-1};P\right)\geq I\left(X_{1};\ldots ;X_{k};P\right)$ (in short: $I_{k-1}\geq I_{k}$),
- $X_{k}.I\left(X_{1};\ldots ;X_{k-1};P\right)<0$ if and only if $I\left(X_{1};\ldots ;X_{k-1};P\right)<I\left(X_{1};\ldots ;X_{k};P\right)$ (in short: $I_{k-1}<I_{k}$),

Which fully characterize the phenomenon of information negativity as an increasing or diverging sequence of mutual information.

### 2.3. Information Structures and Coboundaries

**Figure 1:**
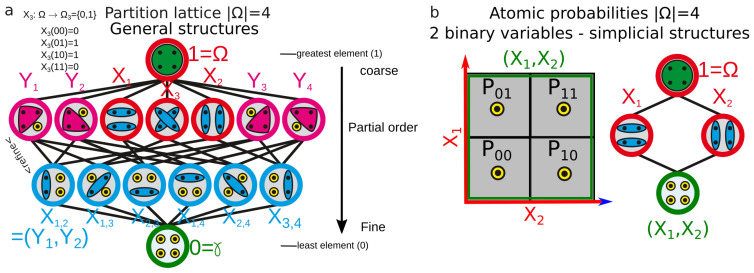
Example of general and simplicial information structures. (**a**) Example of lattice of random variables (partitions): the lattice of partitions of atomic-elementary events for a sample space of four atomic elements |Ω|=4 (e.g., two coins and Ω={00,01,10,11}), each element being denoted by a black dot in the circles representing the random variables. The joint operation of random variables denoted (X,Y) or X⊗Y of two partitions is the less-fine partition *Z* that is finer than *X* and *Y* (*Z* divides *Y* and *X* or *Z* is the greatest common divisor of *Y* and *X*). It is represented by the coincidence of two edges of the lattices. The joint operation has an identity element denoted 1=Ω (that we will denote 0 hereafter), with X,1=X,Ω=X and is idempotent $\left(X,X\right)=X^{2}=X$. The structure is a partially ordered set (poset) with a refinement relation. (**b**) Illustration of the simplicial structure (sublattice) used for the data analysis (|Ω|=4 as previously).

This section justifies the choice of functions and algorithm, the topological nature of the data analysis and the approximations we had to concede for the computation. In the general formulation of information cohomology, the random variables are partitions of the atomic probabilities of a finite probability space (Ω,B,P) (e.g., all their equivalence classes). The Joint-Variable $\left(X_{1},X_{2}\right)$ is the less-fine partition that is finer than $X_{1}$ and $X_{2}$; the whole lattice of partitions Π [98] corresponds to the lattice of joint random variables [1,99]. Then, a general information structure is defined to be the triple (Ω,Π,P). A more modern and general expression in category theory and topos is given in References [1,18]. $\left(X_{1},\ldots ,X_{k};P\right)$ designates the image law of the probability *P* by the measurable function of joint variables $\left(X_{1},\ldots ,X_{k}\right)$. Figure 1 gives a simple example of the lattice of partitions for four atomic probabilities, with the simplicial sublattice used for data analysis. Atomic probabilities are also illustrated in a figure in the associated paper [2].

On this general information structure, we consider the real module of all measurable functions $F(X_{1},\ldots ,X_{k};P)$ and the conditioning-expectation by *Y* of measurable functions as the action of *Y* on the functional module, denoted $Y.F(X_{1},\ldots ,X_{k};P)$, such that it corresponds to the usual definition of conditional entropy (Equation (8)). We define our complexes of measurable functions of random variables $X^{k}=F\left(X_{1},\ldots ,X_{k};P\right)$ and the cochain complexes $\left(X^{k},\partial^{k}\right)$ as:

$$0\to X^{0}\overset{\partial^{0}}{\to }X^{1}\overset{\partial^{1}}{\to }X^{2}\overset{\partial^{2}}{\to }\ldots X^{k-1}\overset{\partial^{k-1}}{\to }X^{k},$$

where $\partial^{k}$ is the left action co-boundary that Hochschild proposed for associative and ring structures [100]. A similar construction of a random variable complex was given by Drumond-Cole, Park and Terilla [84,85]. We also consider the two other directly related cohomologies defined by considering a trivial left action [1] and a symmetric (left and right) action [22,101,102] of conditioning:

- The left action Hochschild-information coboundary and cohomology (with trivial right action):
  (18) $$\begin{array}{cc} \left(\partial^{k}\right)F\left(X_{1};X_{2};\ldots ;X_{k+1};P\right)= & X_{1}.F\left(X_{2};\ldots ;X_{k+1};P\right)\\ \; & +\sum_{i=1}^{k}{\left(-1\right)}^{i}F\left(X_{1};X_{2};\ldots ;\left(X_{i},X_{i+1}\right);\ldots ;X_{k+1};P\right)\\ \; & +{\left(-1\right)}^{k+1}F\left(X_{1};\ldots ;X_{k};P\right). \end{array}$$
  This coboundary, with a trivial right action, is the usual coboundary of Galois cohomology ([103], p. 2) and in general it is the coboundary of homological algebra obtained by Cartan and Eilenberg [104] and MacLane [105] (non-homogenous bar complex).
- The “topological-trivial” Hochschild-information coboundary and cohomology: consider a trivial left action in the preceding setting (e.g., $X_{1}.F\left(X_{2};\ldots ;X_{k+1}\right)=F\left(X_{2};\ldots ;X_{k+1}\right)$). It is the subset of the preceding case, which is invariant under the action of conditioning. We obtain the topological coboundary $\left(\partial_{t}^{k}\right)$ [1]:
  (19) $$\begin{array}{cc} \left(\partial_{t}^{k}\right)F\left(X_{1};X_{2};\ldots ;X_{k+1};P\right)= & F(X_{2};\ldots ;X_{k+1};P)\\ \; & +\sum_{i=1}^{k}{\left(-1\right)}^{i}F\left(X_{1};X_{2};\ldots ;\left(X_{i},X_{i+1}\right);\ldots ;X_{k+1};P\right)\\ \; & +{\left(-1\right)}^{k+1}F\left(X_{1};\ldots ;X_{k};P\right). \end{array}$$
- The symmetric Hochschild-information coboundary and cohomology: as introduced by Gerstenhaber and Shack [22], Kassel [102] (p. 13) and Weibel [101] (chap. 9), we consider a symmetric (left and right) action of conditioning, that is, $X_{1}.F\left(X_{2};\ldots ;X_{k+1}\right)=F\left(X_{2};\ldots ;X_{k+1}\right).X_{1}$. The left action module is essentially the same as considering a symmetric action bimodule [22,101,102]. We hence obtain the following symmetric coboundary $\left(\partial_{*}^{k}\right)$:
  (20) $$\begin{array}{cc} \left(\partial_{*}^{k}\right)F\left(X_{1};X_{2};\ldots ;X_{k+1};P\right)= & X_{1}.F\left(X_{2};\ldots ;X_{k+1};P\right)\\ \; & +\sum_{i=1}^{k}{\left(-1\right)}^{i}F\left(X_{1};X_{2};\ldots ;\left(X_{i},X_{i+1}\right);\ldots ;X_{k+1};P\right)\\ \; & +{\left(-1\right)}^{k+1}X_{k+1}.F\left(X_{1};\ldots ;X_{k};P\right). \end{array}$$

Based on these definitions, Baudot and Bennequin [1] computed the first homology class in the left action Hochschild-information cohomology case and the coboundaries in higher degrees. We introduce here the symmetric case and detail the higher-degree cases by direct specialization of the co-boundary formulas, such that it appears that information functions and chain rules are homological by nature. For notation clarity, we omit the probability in the writing of the functions and when specifically stated replace their notation *F* by their usual corresponding informational function notation H,I.

#### 2.3.1. First Degree (k=1)

For the first degree k=1, we have the following results:

- The left 1-co-boundary is $\left(\partial^{1}\right)F\left(X_{1};X_{2}\right)=X_{1}.F\left(X_{2}\right)-F\left(X_{1},X_{2}\right)+F\left(X_{1}\right)$. The 1-cocycle condition $\left(\partial^{1}\right)F\left(X_{1};X_{2}\right)=0$ gives $F\left(X_{1},X_{2}\right)=F\left(X_{1}\right)+X_{1}.F\left(X_{2}\right)$, which is the chain rule of information shown in Equation (10). Then, following Kendall [106] and Lee [107], it is possible to recover the functional equation of information and to characterize uniquely—up to the arbitrary multiplicative constant *k*—the entropy (Equation (1)) as the first class of cohomology [1,18]. This main theorem allows us to obtain the other information functions in what follows. Marcolli and Thorngren [20] and the group of Leinster, Fritz and Baez [81,82] independently obtained an analog result using a measure-preserving function and a characteristic one Witt construction, respectively. In these various theoretical settings, this result extends to relative entropy [1,20,82] and Tsallis entropies [18,20].
- The topological 1-coboundary $\left(\partial_{t}^{1}\right)$ is $\left(\partial_{t}^{1}\right)F\left(X_{1};X_{2}\right)=F\left(X_{2}\right)-F\left(X_{1},X_{2}\right)+F\left(X_{1}\right)$, which corresponds to the definition of mutual information $\left(\partial_{t}^{1}\right)F\left(X_{1};X_{2}\right)=I\left(X_{1};X_{2}\right)=H\left(X_{1}\right)+H\left(X_{2}\right)-H\left(X_{1},X_{2}\right)$ and hence $I_{2}$ is a topological 1-coboundary.
- The symmetric 1-coboundary $\left(\partial_{*}^{1}\right)$ is $\left(\partial_{*}^{1}\right)F\left(X_{1};X_{2}\right)=X_{1}.F\left(X_{2}\right)-F\left(X_{1},X_{2}\right)+X_{2}.F\left(X_{1}\right)$, which corresponds to the negative of the pairwise mutual information $\left(\partial_{*}^{1}\right)F\left(X_{1};X_{2}\right)=X_{2}.H\left(X_{1}\right)+X_{1}.H\left(X_{2}\right)-H\left(X_{1},X_{2}\right)=-I\left(X_{1};X_{2}\right)$ and hence $-I_{2}$ is a symmetric 1-coboundary. Moreover, the 1-cocycle condition $\left(\partial_{*}^{1}\right)F\left(X_{1};X_{2}\right)=0$ characterizes functions satisfying $F\left(X_{1},X_{2}\right)=X_{2}.F\left(X_{1}\right)+X_{1}.F\left(X_{2}\right)$, which corresponds to the information pseudo-metric discovered by Shannon [23], Rajski [24], Zurek [25] and Bennett [26] and has further been applied for hierarchical clustering and finding categories in data by Kraskov and Grassberger [27]: $H\left(X_{1}△X_{2}\right)=X_{2}.H\left(X_{1}\right)+X_{1}.H\left(X_{2}\right)=H\left(X_{1},X_{2}\right)-I\left(X_{1};X_{2}\right)$. Therefore, up to an arbitrary scalar multiplicative constant *k*, the information pseudo-metric $H(X_{1}△X_{2})$ is the first class of symmetric cohomology. This pseudo-metric is represented in Figure 3. It generalizes to pseudo *k*-volumes that we define by $V_{k}=H_{k}-I_{k}$ (particularly interesting symmetric nonnegative functions computed by the provided software).

#### 2.3.2. Second Degree (k=2)

For the second degree k=2, we have the following results:

- The left 2-co-boundary is $\partial^{2}F\left(X_{1};X_{2};X_{3}\right)=X_{1}.F\left(X_{2};X_{3}\right)-F\left(\left(X_{1},X_{2}\right);X_{3}\right)+F\left(X_{1};\left(X_{2},X_{3}\right)\right)-F\left(X_{1};X_{2}\right)$, which corresponds to minus the 3-mutual information $\partial^{2}F\left(X_{1};X_{2};X_{3}\right)=X_{1}.I\left(X_{2};X_{3}\right)-I\left(\left(X_{1},X_{2}\right);X_{3}\right)+I\left(X_{1};\left(X_{2},X_{3}\right)\right)-I\left(X_{1};X_{2}\right)=-I\left(X_{1};X_{2};X_{3}\right)$ and hence $-I_{3}$ is the left 2-coboundary.
- The topological 2-coboundary is $\left(\partial_{t}^{2}\right)F\left(X_{1};X_{2};X_{3}\right)=F\left(X_{2};X_{3}\right)-F\left(\left(X_{1},X_{2}\right);X_{3}\right)+F\left(X_{1};\left(X_{2},X_{3}\right)\right)-F\left(X_{1};X_{2}\right)$, which corresponds in information to $\partial_{t}^{2}F\left(X_{1};X_{2};X_{3}\right)=I\left(X_{2};X_{3}\right)-I\left(\left(X_{1},X_{2}\right);X_{3}\right)+I\left(X_{1};\left(X_{2},X_{3}\right)\right)-I\left(X_{1};X_{2}\right)=0$ and hence the topological 2-coboundary is always null-trivial.
- The symmetric 2-coboundary is $\left(\partial_{*}^{2}\right)F\left(X_{1};X_{2};X_{3}\right)=X_{1}.F\left(X_{2};X_{3}\right)-F\left(\left(X_{1},X_{2}\right);X_{3}\right)+F\left(X_{1};\left(X_{2},X_{3}\right)\right)-X_{3}.F\left(X_{1};X_{2}\right)$, which corresponds in information to $\partial_{*}^{2}F\left(X_{1};X_{2};X_{3}\right)=X_{1}.I\left(X_{2};X_{3}\right)-I\left(\left(X_{1},X_{2}\right);X_{3}\right)+I\left(X_{1};\left(X_{2},X_{3}\right)\right)-X_{3}.I\left(X_{1};X_{2}\right)=0$ and hence the symmetric 2-coboundary is always null-trivial.

#### 2.3.3. Third Degree (k=3)

For the third degree k=3, we have the following results:

- The left 3-co-boundary is $\partial^{3}F\left(X_{1};X_{2};X_{3};X_{4}\right)=X_{1}.F\left(X_{2};X_{3};X_{4}\right)-F\left(\left(X_{1},X_{2}\right);X_{3};X_{4}\right)+F\left(X_{1};\left(X_{2},X_{3}\right);X_{4}\right)-F\left(X_{1};X_{2};\left(X_{3},X_{4}\right)\right)+F\left(X_{1};X_{2};X_{3}\right)$, which corresponds in information to $\partial^{3}F\left(X_{1};X_{2};X_{3};X_{4}\right)=X_{1}.I\left(X_{2};X_{3};X_{4}\right)-I\left(\left(X_{1},X_{2}\right);X_{3};X_{4}\right)+I\left(X_{1};\left(X_{2},X_{3}\right);X_{4}\right)-I\left(X_{1};X_{2};\left(X_{3},X_{4}\right)\right)+I\left(X_{1};X_{2};X_{3}\right)=0$ and hence the left 3-coboundary is always null-trivial.
- The topological 3-coboundary is $\partial_{t}^{3}F\left(X_{1};X_{2};X_{3};X_{4}\right)=F\left(X_{2};X_{3};X_{4}\right)-F\left(\left(X_{1},X_{2}\right);X_{3};X_{4}\right)+F\left(X_{1};\left(X_{2},X_{3}\right);X_{4}\right)-F\left(X_{1};X_{2};\left(X_{3},X_{4}\right)\right)+F\left(X_{1};X_{2};X_{3}\right)$, which corresponds in information to $\partial_{t}^{3}F\left(X_{1};X_{2};X_{3};X_{4}\right)=I\left(X_{2};X_{3};X_{4}\right)-I\left(\left(X_{1},X_{2}\right);X_{3};X_{4}\right)+I\left(X_{1};\left(X_{2},X_{3}\right);X_{4}\right)-I\left(X_{1};X_{2};\left(X_{3},X_{4}\right)\right)+I\left(X_{1};X_{2};X_{3}\right)=I\left(X_{1};X_{2};X_{3};X_{4}\right)$ and hence $I_{4}$ is a topological 3-coboundary.
- The symmetric 3-coboundary is $\left(\partial_{*}^{3}\right)F\left(X_{1};X_{2};X_{3};X_{4}\right)=X_{1}.F\left(X_{2};X_{3};X_{4}\right)-F\left(\left(X_{1},X_{2}\right);X_{3};X_{4}\right)+F\left(X_{1};\left(X_{2},X_{3}\right);X_{4}\right)-F\left(X_{1};X_{2};\left(X_{3},X_{4}\right)\right)+X_{4}.F\left(X_{1};X_{2};X_{3}\right)$, which corresponds in information to $\partial_{*}^{3}F\left(X_{1};X_{2};X_{3};X_{4}\right)=X_{1}.I\left(X_{2};X_{3};X_{4}\right)-I\left(\left(X_{1},X_{2}\right);X_{3};X_{4}\right)+I\left(X_{1};\left(X_{2},X_{3}\right);X_{4}\right)-I\left(X_{1};X_{2};\left(X_{3},X_{4}\right)\right)+X_{4}.I\left(X_{1};X_{2};X_{3}\right)=-I\left(X_{1};X_{2};X_{3};X_{4}\right)$ and hence $-I_{4}$ is a symmetric 3-coboundary.

#### 2.3.4. Higher Degrees

For k=4, we obtain $\partial^{4}F\left(X_{1};X_{2};X_{3};X_{4};X_{5}\right)=-I_{5}$ and $\partial_{t}^{5}F\left(X_{1};X_{2};X_{3};X_{4};X_{5}\right)=0$ and $\partial_{*}^{5}F\left(X_{1};X_{2};X_{3};X_{4};X_{5}\right)=0$. For arbitrary *k*, the symmetric coboundaries are just the opposite of the topological coboundaries $\partial_{t}^{k}=-\partial_{*}^{k}$. It is possible to generalize to arbitrary degrees [1] by remarking that:

- For even degrees 2k: we have $I_{2k}=-\partial_{t}I_{2k-1}$, and then $I_{2k}=\partial_{t}\partial \partial_{t}\ldots \partial \partial_{t}H$ with 2k−1 boundary terms. In conclusion, we have:
  (21) $$\partial^{2k}F=-I_{2k+1}\;\;\mathrm{and}\;\;\partial_{*}^{2k}F=-\partial_{t}^{2k}F=0.$$
- For odd degrees 2k+1: $I_{2k+1}=-\partial I_{2k-1}$ and then $I_{2k+1}=-\partial \partial_{t}\partial \ldots \partial \partial_{t}H$ with 2k boundary terms. In conclusion, we have:
  (22) $$\partial^{2k-1}F=0\;\;\mathrm{and}\;\;\partial_{*}^{2k-1}F=-\partial_{t}^{2k}F=-I_{2k}.$$

In References [2,108] (Theorem 2), we show that the mutual independence of *n* variables is equivalent to the vanishing of all $I_{k}$ functions for all 2≤k≤n. As a probabilistic interpretation and conclusion, the information cohomology hence quantifies statistical dependences at all degrees and the obstruction to factorization. Moreover, *k*-independence coincides with cocycles. We therefore expect that the higher cocycles of information, conjectured to be polylogarithmic forms [1,77,78], are characterized by the functional equations $I_{k}=0$ and quantify statistical *k*-independence.

## 3. Simplicial Information Cohomology

### 3.1. Simplicial Substructures of Information

The general information structure, relying on the information functions defined on the whole lattice of partitions, encompasses all possible statistical dependences and relations, since by definition it considers all possible equivalent classes on a probability space. One could hence expect this general structure to provide a promising theoretical framework for classification tasks on data and this is probably true in theory. However, this general case hardly allows any interesting computational investigation, as it implies an exhaustive exploration of computational complexity following Bell’s combinatoric in $O(exp\left(exp\left(N^{n}\right)\right)$) for *nN*-ary variables. This fact was already remarked in the study of aggregation for artificial intelligence by Lamarche-Perrin and colleagues [109]. At each order *k*, the number of *k*-joint-entropy and *k*-mutual-information to evaluate is given by Stirling numbers of the second kind S(n,k) that sum to Bell number $B_{n}$, $B_{n}=\sum_{k=0}^{n}S\left(n,k\right)$. For example, considering 16 variables that can take 8 values each, we have $8^{16}=2^{48}\approx 3.10^{14}$ atomic probabilities and the partition lattice of variables exhibits around $e^{e^{2^{48}}-1}\geq 2^{200}$ elements to compute. This computational reef can be decreased by considering the sample size *m*, which is the number of trials, repetitions or points used to effectively estimate the empirical probability. It restricts the computation to O(exp(exp(m)), which remains insurmountable in practice with our current classical Turing machines. To circumvent this computational barrier, data analysis is developed on the simplest and oldest subcase of Hochschild cohomology—the simplicial cohomology, which we hence call the simplicial information cohomology and structure and which corresponds to a subcase of cohomology and structure introduced previously (see Figure 1b). It corresponds to Examples 1 and 4 in Reference [1], and to the python scripts shared on Github (cf. Supplementary Materials). For simplicity, we note also the simplicial information structure $\left(\Omega ,\Delta^{n},P\right)$, $\Delta^{n}=\left(X_{1},\ldots ,X_{n};P\right)$, as we will not come back to the general setting. Joint $\left(X_{1},X_{2}\right)$ and meet $\left(X_{1};X_{2}\right)$ operations on random variables are the usual joint-union and meet-intersection of Boolean algebra and define two opposite-dual monoids, freely generating the lattice of all subsets and its dual. The combinatorics of the simplicial information structure follow binomial coefficients and, for each degree *k* in an information structure of *n* variables, we have nk=n!k!(n−k!) elements that are in one-to-one correspondence with the *k*-faces (the *k*-tuples) of the *n*-simplex of random variables (or its barycentric subdivisions). It is a (simplicial) substructure of the general structure, since any finite lattice is a sub-lattice of the partition lattice [110]. This lattice embedding and the fact that simplicial cohomology is a special case of Hochschild cohomology can also be inferred directly from their coboundary expression and has been explicitly formalized in homology: notably, Gerstenhaber and Shack showed that a functor, denoted $\Sigma \mapsto k_{\Sigma }!$, induces an isomorphism between simplicial and Hochschild cohomology $H^{•}\left(\Sigma ,k\right)≅H^{•}\left(k_{\Sigma }!,k_{\Sigma }!\right)$ (see [111] for precisions). A simplicial complex $X^{k}=F\left(X_{1},\ldots ,X_{k};P\right)$ of measurable functions is any subcomplex of this simplex $\Delta^{n}$ with k≤n and any simplicial complex can be realized as a subcomplex of a simplex (see p. 296 in Reference [112]). The information landscapes presented in Section 3.3 illustrate an example of such a lattice/information structure. Moreover in this ordinary homological structure, the degree obviously coincides with the dimension of the data space (the data space is in general $R^{n}$, the space of “co-ordinate” values of the variables). This homological (algebraic, geometric and combinatorial) restriction to the simplicial subcase can have some important statistical consequences. In practice, whereas the consideration of the partition lattice ensured that no reasonable (up to logical equivalence) statistical dependences could be missed (since all the possible equivalence classes on the atomic probabilities were considered), the monoidal simplicial structure unavoidably misses some possible statistical dependences, as shown and exemplified by James and Crutchfield [51].

### 3.2. Topological Self and Free Energy of *K*-Body Interacting System-Poincaré-Shannon Machine

#### Topological Self and Free Energy of *K*-Body Interacting Systems

The basic idea behind the development of topological quantum field theories [113,114,115] was to define the action and energy functionals on a purely topological ground, independently of any metric assumptions and to derive from this the correlation functions or partition functions. Here, in an elementary model for applied purposes, we define, in the special case of classical and discrete probability, the *k*-mutual information $I_{k}$ (that generalize the correlation functions to nonlinear relation [116]), as the contribution of the *k*-body interactions to the energy functional. Some further observations support such a definition: (i) as stated in Reference [1], the signed mutual information ${\left(-1\right)}^{k}I_{k}$ defining energy are sub-harmonic, a kind of weak convexity; (ii) in the next sections, we define the paths of information and show that they are equivalent to the discrete symmetry group; (iii) from the empirical point of view, the results in Section 3.4.5 shows that these energy functionals estimated on real data behave as expected for usual *k*-body homogeneous formalism such as the van der Waals model or more refined density functional theory (DFT) [117,118]. Given in the context of simplicial structures, these definitions generalize to the case of a partitions lattice and altogether provide the usual thermodynamical and machine-learning expressions and interpretation of mutual information quantities: some new methods free of metric assumptions. There are two qualitatively and formally different components in the $I_{k}$, that give the two following definitions.

**Definition** **1.** *Self-internal energy (definition): for k=1, $I_{1}$ and their sum in an information structure expressed in Equation (16), namely, $\sum_{T\subset [n];card(T)=1}I_{1}\left(X_{T};P\right)$, are a self-interaction component, since they sum over marginal information entropy $I_{1}\left(X_{i}\right)=H_{1}\left(X_{i}\right)$. We call the first-dimension mutual-information component $U(X_{1},\ldots ,X_{n};P_{N})$ the self-information or internal energy, in analogy to usual statistical physics and notably DFT:*

(23) $$U\left(X_{1},\ldots ,X_{n};P_{N}\right)=\sum_{i=1}^{n}I_{1}\left(X_{i};P_{N}\right).$$

Note that in the present context, which is discrete and where the interactions do not depend on a metric, the self-interaction does not diverge, which is a usual problem with metric continuous formalism and was the original motivation for regularization and renormalization infinite corrections, considered by Feynman and Dirac as the mathematical default of the formalism [44,45].

**Definition** **2.** *k-free-energy and total-free-energy: for k≥2, ${\left(-1\right)}^{k}I_{k}$ and their sum in an information structure (Equation 16) quantify the contribution of the k-body interactions. We call the kth dimension mutual-information component ${\left(-1\right)}^{k}I_{k}$ given in Equation (5) the k-free-information-energy. We call the (cumulative) sum over dimensions of these k-free-information-energies starting at pairwise interactions (dimension 2), the total n-free-information-energy and denote it $G(X_{1},\ldots ,X_{n};P_{N})$:*

(24) $$G\left(X_{1},\ldots ,X_{n};P_{N}\right)=\sum_{i=2}^{n}{\left(-1\right)}^{i-1}\sum_{I\subset [n];card(I)=i}I_{i}\left(X_{I};P_{N}\right)=C_{n}\left(X_{1};\ldots X_{n};P_{N}\right).$$

The total free energy is the total correlation (Equation (7)) introduced by Watanabe in 1960 [94] that quantifies statistical dependence in the work of Studený and Vejnarova [96] and Margolin and colleagues [97] and among other examples consciousness in the work of Tononi and Edelman [95]. In agreement with the results of Baez and Pollard in their study of biological dynamics using out-of-equilibrium formalism [32] and the appendix of the companion paper on Bayes free energy [2], the total free energy is a relative entropy. The consideration that free energy is the peculiar case of total correlation within the set of relative entropies accounts for the fact that the free energy shall be a symmetric function of the variables associated to the various bodies (e.g., f(X;Y)=f(Y;X) in the pairwise interaction case). Moreover, whereas the $I_{k}$ energy component can be negative, the $G_{k}$ total energy component is always non-negative. Each ${\left(-1\right)}^{k}I_{k}$ term in the free energy can be understood as a free-energy correction accounting for the *k*-body interactions.

Entropy is given by the alternated sums of information (Equation (16)), which then read as the usual isotherm thermodynamic relation:

(25) $$H_{n}\left(X_{1},\ldots ,X_{n};P_{N}\right)=U\left(X_{1},\ldots ,X_{n};P_{N}\right)-G\left(X_{1},\ldots ,X_{n};P_{N}\right).$$

This information-theoretic formulation of thermodynamic relation follows Jaynes [9,10], Landauer [11], Wheeler [119] and Bennett’s [14] original work and is general in the sense that it is finite and discrete and holds independently of the assumption of the system being in equilibrium or not (i.e., for any finite probability). In more probabilistic terms, it does not assume that the variables are identically distributed—a required condition for the application of classical central limit theorems (CLTs) to obtain the normal distributions in the asymptotic limit [120]. In the special case where one postulates that the probability follows the equilibrium Gibbs distribution, which is also the maximum entropy distribution [121,122], the expression of the joint entropy (k=−1/ln2) allows recovery of the equilibrium fundamental relation, as usually achieved in statistical physics (see Adami and Cerf [123] and Kapranov [124] for more details). Explicitly, let us consider Gibbs’ distribution:

(26) $$p\left(X_{1}=x_{1},\ldots ,X_{n}=x_{n}\right)=p_{\underset{n\;\mathrm{indices}}{\underset{︸}{ij\ldots n}}}=\frac{1}{Z}e^{-\beta E_{ij\ldots n}/k_{B}T},$$

where $E_{ij\ldots n}$ is the energy of the elementary-atomic probability $p_{ij\ldots n}$, $k_{B}$ is Boltzmann’s constant, *T* is the temperature and $Z=\sum_{i,j,\ldots ,n}^{N_{i}.N_{j}\ldots N_{n}}e^{-E_{ij\ldots n}/k_{B}T}$ is the partition function, such that $\sum_{i,j,\ldots ,n}^{N_{i}.N_{j}\ldots N_{n}}p_{ij\ldots n}=1$. Since $H\left(X_{1},\ldots ,X_{n}\right)=k\sum_{i,j,\ldots ,n}^{N_{i}.N_{j}\ldots N_{n}}p_{ij\ldots n}\ln p_{ij\ldots n}$ equals the thermodynamic entropy function *S* up to the arbitrary Landauer constant factor $k_{B}\ln2$, $S=k_{B}\ln2H\left(X_{1},\ldots ,X_{n}\right)$, the entropy for the Gibbs distribution gives:

(27) $$H\left(X_{1},\ldots ,X_{n}\right)/k=\sum_{i,j,\ldots ,n}^{N_{1}.N_{2}\ldots N_{n}}p_{ij\ldots n}E_{ij\ldots n}/k_{B}T+\sum_{i,j,\ldots ,n}^{N_{1}.N_{2}\ldots N_{n}}p_{ij\ldots n}\ln Z=\left(\langle E\rangle -G\right)/k_{B}T,$$

which gives the expected thermodynamical relation:

(28) $$k_{B}T\ln2.H\left(X_{1},\ldots ,X_{n}\right)=\langle E\rangle -G=U-G,$$

where *G* is the free energy $G=-k_{B}T\ln Z$.

In the general case of arbitrary random variables (not necessarily i.i.d.) and discrete probability space, the identification of marginal information with internal energy

(29) $$\sum_{k=1}^{n}H\left(X_{k}\right)=\sum_{i,j,\ldots ,n}^{N_{1}.N_{2}\ldots N_{n}}p_{ij\ldots n}E_{ij\ldots n}$$

implies by direct algebraic calculus that:

(30) $$\sum_{i,j,\ldots ,n}^{N_{1}.N_{2}\ldots N_{n}}p_{ij\ldots n}E_{ij\ldots n}=-\sum_{i,j,\ldots ,n}^{N_{1}.N_{2}\ldots N_{n}}p_{ij\ldots n}\ln\left(\prod_{k=i}^{n}p_{••\ldots k\ldots •}\right),$$

where the marginal probability $p_{••\ldots k\ldots •}$ is the sum over all probabilities for which $X_{k}=x_{k}$. It is hence tempting to identify the elementary atomic energies $E_{ij\ldots n}$ with the elementary marginal information $\ln p_{••\ldots k\ldots •}$. This is achieved uniquely by considering that such an elementary energy function must satisfy the additivity axiom (extensivity): ($E\left(X_{i}=x_{i},Xj=x_{j}\right)=E_{i,j}=E_{ij}=E_{i}+E_{j}$), which is the functional equation of the logarithm. The original proof goes back at least to Kepler, an elementary version was given by Erdos [125] and in information theoretic terms can be found in the proofs of uniqueness of “single event information function” by Aczel and Darokzy ([126], p. 3). It establishes the following proposition:

**Theorem** **1.** *Given a simplicial information structure, the elementary energies satisfying the extensivity axiom are the functions:*

(31) $$E_{ij\ldots n}=k\sum_{k=i}^{n}\ln p_{••\ldots k\ldots •},$$

*where k is an arbitrary constant settled to k=−1/ln2 for units in bits.*

**Figure 2:**
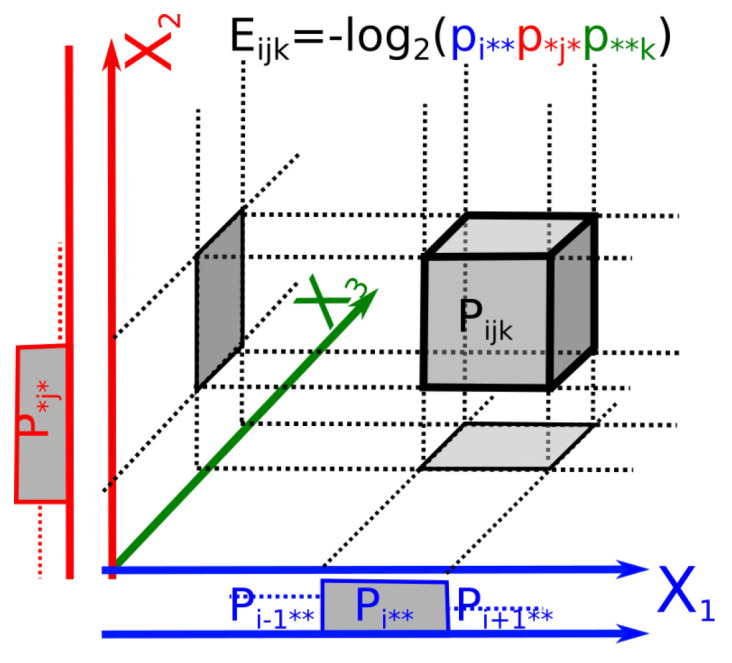
Elementary energy as logarithm of locally Euclidean probability volumes. Example of an elementary energy $E_{ijk}$ associated to a probability $p_{ijk}$ (n=3 variables). The histograms of the marginal distributions of each variable are plotted beside the axes.

The geometric meaning of these elementary energies as log of marginal elementary probability volumes (locally Euclidean) is illustrated in Figure 2 and further underlines that $I_{k,k\geq 2}$ are volume corrections accounting for the statistical dependences among marginal variables.

**Examples:** (i) In the example of three binary random variables ($n=3,N_{1}=N_{2}=N_{3}=2$, three variables of Bernoulli) illustrated in the figure of the associated paper [2], we have $E_{000}=-\ln\left(p_{0••}p_{•0•}p_{••0}\right)$, $E_{000}=-\ln\left(p_{000}+p_{010}+p_{001}+p_{011}\right)-\ln\left(p_{000}+p_{100}+p_{001}+p_{101}\right)-\ln\left(p_{000}+p_{100}+p_{010}+p_{110}\right)$ and in the configuration of negative-entangled-Borromean information of the figure of the associated paper [2], we obtain $E_{000}=3$ in bit units and similarly $E_{001}=E_{010}=E_{011}=E_{101}=E_{110}=E_{111}=3$ and we hence recover $U=\sum_{i,j,k}^{8}p_{ijk}E_{ijk}=\sum_{i=1}^{3}H\left(X_{i}\right)=3$ bits. Note that the limit 0ln0∼0 avoids singularity of elementary energies.

(ii) In the special case of identically distributed variables, $p_{••\ldots k\ldots •}=p_{•\ldots j\ldots ••}$, we have $E_{ij\ldots n}=nk\ln p_{••\ldots k\ldots •}$, and hence the marginal Gibbs distribution:

(32) $$p_{••\ldots k\ldots •}=e^{\frac{E_{ij\ldots n}}{nk}}.$$

(iii) For independent identically distributed variables (non-interacting), we have $G_{n}=0$ and hence:

(33) $$H_{n}\left(X_{1},\ldots ,X_{n};P_{N}\right)=U\left(X_{1},\ldots ,X_{n};P_{N}\right)=nH\left(X_{i}\right).$$

(iv) Considering the variables to be the 6n variables of the phase space, with one variable of position and one variable of momentum per body (denoted $\left(X_{k}^{1},X_{k}^{2},X_{k}^{3},P_{k}^{1},P_{k}^{2},P_{k}^{3}\right)$ for the *k*th body), it is possible to re-express the semi-classical formalism, according to which the entropy formulation is (p. 22, [127]):

(34) $$H_{6n}\left(X_{1}^{1},X_{1}^{2},X_{1}^{3},P_{1}^{1},P_{1}^{2},P_{1}^{3},\ldots ,P_{n}^{3};P_{N}\right)=\log\left(\frac{\Delta X\Delta P}{{\left(2\pi \hbar \right)}^{6n}}\right).$$

This is achieved by identifying the internal and free energy as follows:

(35) 〈E〉=−6nlog(2πℏ),

(36) G=−log(ΔXΔP).

This identifies the elementary volumes/probabilities with the Planck constant, the quantum of action (the consistency in the units is realized in Section 3.4.3 by the introduction of time). The quantum of action can be illustrated by considering in Figure 2 that it is the surface of the square/rectangle for two conjugate variables (considered as position and momentum). In this setting, ΔXΔP quantifies the non-extensivity of the volume in the phase-space due to interactions or in other words, the mutual information accounts for the consideration of the dependence of the subsystems considered as opened and exchanging energy. As noted by Baez and Pollard, the relative entropy provides a quantitative measure of how far from equilibrium the whole system is [32]. The basic principle of this expression of information theory in physics has been known at least since Jaynes’s work [9,10].

As a conclusion, information topology applies—without imposing metric, symplectic or contact structures—to the physical formalism of *n*-body interacting systems relying on empirical measures. Considering the 3n or 6n dimensions (degrees of freedom) of a configuration or a phase space as random variables, it is possible to recover the (semi)classical statistical physics formalism. It is also interesting to discuss the status of the analog of the temperature variable in the present formalism which is played by the graining, which is the size $N_{i}$ of the alphabet of a variable $X_{i}$. In usual thermodynamics we have $H\left(X^{n};P_{N}\right)=T.S\left(X^{n}\right)$ and to stay consistent, temperature shall be a functional inverse of the graining *N*, the lowest temperature being the finest grain (large *N*) and the highest temperature being the coarsest grain (small *N*).

### 3.3. *k*-Entropy and *k*-Information Landscapes

**Definition** **3.** *Information Landscapes: Information landscapes are a representation of the (semi)lattice of information structures where each element is represented as a function of its corresponding value of entropy or mutual information. In abscissa are the dimensions k and in ordinate the values of the information functions of a given subset of k variables.*

**Figure 3:**
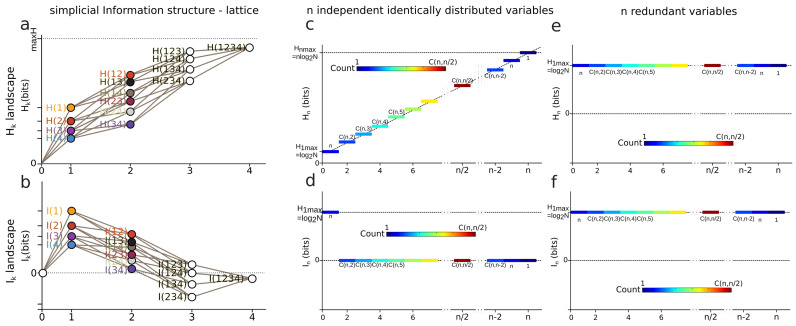
Entropy and information landscapes. (**a**) Illustration of the principle of an entropy $H_{k}$ landscape and (**b**) of a mutual information $I_{k}$ landscape for n=4 random variables. The lattice of the simplicial information structure is depicted with grey lines. Theoretical examples of entropy and information landscapes. (**c**,**d**) $H_{k}$ and $I_{k}$ landscapes for *n* independent and identically distributed variables. The degeneracy of $H_{k}$ and $I_{k}$ values is represented by a color code: the number of *k*-tuples having the same information value. (**e**,**f**) $H_{k}$ and $I_{k}$ landscapes for *n* fully redundant variables. Such variables are equivalent from the information point of view; they are identically distributed and fully dependent.

In data science terms, these landscapes provide a visualization of the potentially high-dimensional structure of the data points. In information theoretic terms, it provides a representation of Shannon’s work on lattices [23], further developed by Han [128]. $H_{k}$ and $I_{k}$, as real continuous functions, provide a ranking of the lattices at each dimension *k*. It is the ranking (i.e., the relative values of information) which matters and comes out of the homological approach, rather than the absolute values. The principle of $H_{k}$ and $I_{k}$ landscapes is illustrated in Figure 3 for n=4. $H_{k}$ and $I_{k}$ analyze and quantify the variability-randomness and statistical dependences at all dimensions *k*, respectively, from 1 to *n*, *n* being the total number of variables under study. The $H_{k}$ landscape represents the values of joint entropy for all *k*-tuples of variables as a function of the dimensions *k*, the number of variables in the *k*-tuple, together with the associated edges–paths of the lattice (in grey). The $I_{k}$ landscape represents the values of mutual information for all *k*-tuples of variables as a function of the dimension *k*, which is the number of variables in the *k*-tuple. Figure 3 gives two theoretical extremal examples of such landscapes: one for independent and identically distributed variables (totally disordered) and one for fully dependent identically distributed variables (totally ordered). The degeneracy of $H_{k}$ and $I_{k}$ values is given by the binomial coefficient (color code in Figure 3), hence allowing one to derive the normal exact expression of the information landscapes in the asymptotic infinite dimensional limit (n→∞) by application of Laplace–Lemoivre theorem. These are theoretical extremal examples: $H_{k}$ and $I_{k}$ landscapes effectively computed and estimated on biological data with a finite sample are shown in References [2,3,108] and in practice the finite sample size (*m*) may impose some bounds on the landscapes.

### 3.4. Information Paths and Minimum Free Energy Complex

In this section we establish that information landscapes and paths directly encode the basic equalities, inequalities and functions of information theory and allow us to obtain the minimum free energy complex that we estimate on data.

#### 3.4.1. Information Paths (Definition)

**Definition** **4.** *Information Paths: On the discrete simplicial information lattice $\Delta_{k}$, we define a path of degree k as a sequence of edges of the lattice that begins at the least element of the lattice (the identity constant “0”), travels along edges from vertex to vertex of increasing dimension and ends at the greatest element of the lattice of dimension k. Information paths are defined on both joint-entropy and meet-mutual-information semi-lattices and the usual joint-entropy and mutual-information functions are defined on each element of such paths. The entropy path and information path of degree k are denoted $HP_{k}$ and $IP_{k}$, respectively and the set of all information paths is denoted ${\mathrm{HP}}_{k}={\left\{HP_{i}\right\}}_{i\in 1,\ldots ,k!}$ for the entropy paths and ${\mathrm{IP}}_{k}={\left\{IP_{i}\right\}}_{i\in 1,\ldots ,k!}$ for the mutual-information paths.*

We have the theorem:

**Theorem** **2.** *The two sets of all information paths ${\mathrm{HP}}_{k}$ and ${\mathrm{IP}}_{k}$ in the simplicial information structure $\Delta_{k}$ are both in bijection with the symmetric group $S_{k}$. Notably, there are k! information paths in $\Delta_{k}$.*

**Proof.**  by simple enumeration, an edge of dimension *m* connects k−m edges of dimension m+1, the number of paths is hence (k−0).(k−1)….(k−k+2).(k−k+1)=k!, hence the conclusion. □

A given path can be identified with a permutation or a total order by extracting the missing variable in a previous node when increasing the dimension, for example the mutual-information path in $\Delta_{4}$: $IP_{i}=0\to \left(0,X_{2}\right)\to \left(0,X_{1},X_{2}\right)\to \left(X_{1},X_{2},X_{4}\right)\to \left(0,X_{1},X_{2},X_{3},X_{4}\right)$ can be noted as the permutation σ:

(37) $$\left(\begin{array}{ccccc} 0 & 1 & 2 & 3 & 4\\ 0 & 2 & 1 & 4 & 3 \end{array}\right)\;\mathrm{or}\;\left(01234\right)\overset{\sigma }{\to }\left(02143\right).$$

We note an information path with arrows, giving for the previous example $IP_{i}=\left(0\to X_{2}\to X_{1}\to X_{4}\to X_{3}\right)$. These paths shall be seen as the automorphisms of {1,2…k}=[k] and the space of entropy and mutual-information paths can be endowed with the structure of two opposite symmetric groups $S_{k}$ and $S_{k}^{opp}$. The equivalence of the set of paths and symmetric group only holds for the subcase of simplicial structures and the information paths in the lattice of partition are obviously much richer. More precisely, the subset of simplicial information paths in the lattice of partitions corresponds to the automorphisms of the lattice. It is known that the finite symmetric group is the automorphism group of the finite partition lattice [129]. The geometrical realization of information paths ${\mathrm{IP}}_{k}$ and ${\mathrm{HP}}_{k}$ consists of two dual permutohedra (see Postnikov [130]) and gives the informational version of the work of Matúš on conditional probability and permutohedra [131].

#### 3.4.2. Derivatives, Inequalities and Conditional Mutual-Information Negativity

##### Derivatives of information paths:

In the information landscapes, the paths $HP_{i}$ and $IP_{i}$ are piecewise linear functions $IP_{i}\left(k\right)$ with $IP_{i}\left(k\right)=I_{k}$, where $I_{k}$ is the mutual information of the *k*-tuple of variables pertaining to the path $IP_{i}$. We define the first derivatives of the paths for both entropy and mutual-information structures as piecewise linear functions:

First derivative of entropy path: the first derivative of an entropy path $HP_{i}\left(k\right)$ is the conditional information $\left(X_{1},\ldots ,X_{k-1}\right).H\left(X_{k};P\right)$:

(38) $$\frac{dHP_{i}\left(k\right)}{dk}=H\left(X_{1},\ldots ,X_{k};P\right)-H\left(X_{1},\ldots ,X_{k-1};P\right)=\left(X_{1},\ldots ,X_{k-1}\right).H\left(X_{k};P\right).$$

**Figure 4:**
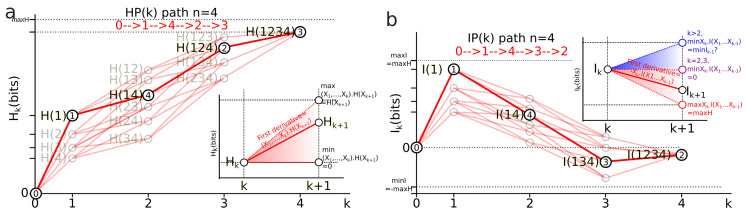
Entropy and information paths. Illustration of an entropy path $HP_{i}=0\to 1\to 4\to 2\to 3$ (**a**) and of a mutual-information path $IP_{i}=0\to 1\to 4\to 3\to 2$ (**b**) for n=4 random variables (see text).

This derivative is illustrated in the graph of Figure 4a. It implements the chain rule of entropy $H_{k+1}-H_{k}=\left(X_{1};\ldots ;\hat{X_{i}};\ldots ;X_{k+1}\right).H\left(X_{i}\right)$ (Equation (12)) and in homology provides a diagram where conditional entropy is a simplicial coface map $\left(X_{1};\ldots ;\hat{X_{i}};\ldots ;X_{k+1}\right).H\left(X_{i}\right)=d^{i}:\;X^{k}\to X^{k+1}$, as a simplicial special case of Hochschild coboundaries (Section 2.3).

First derivative of mutual-information path: the first derivative of an information path $IP_{i}\left(k\right)$ is minus the conditional information $\left(X_{k}\right).I\left(X_{1},\ldots ,X_{k-1};P\right)$:

(39) $$\frac{dIP_{i}\left(k\right)}{dk}=I\left(X_{1},\ldots ,X_{k};P\right)-I\left(X_{1},\ldots ,X_{k-1};P\right)=-X_{k}.I\left(X_{1},\ldots ,X_{k-1};P\right).$$

This derivative is illustrated in the graph of Figure 4b. It implements the chain rule of mutual information $I_{k-1}-I_{k}=X_{k}.I_{k-1}$ (Equation (13)) and in homology provides a diagram where minus the conditional mutual information is a simplicial coface map $X_{i}.I\left(X_{1};\ldots ;\hat{X_{i}};\ldots ;X_{k+1}\right)=d^{i}:\;X^{k}\to X^{k+1}$, introduced in Section 2.3.

##### Bounds of the derivatives and information inequalities

The slope of entropy paths is bounded by the usual conditional entropy bounds ([92] pp. 27–28). Its minimum is 0 and is achieved in the case where $X_{k+1}$ is a deterministic function of ($X_{1},\ldots ,X_{k}$) (lower dashed red line in Figure 4a). Its global upper bound is $\max H_{k+1}=k.\ln\left(N_{1}\ldots N_{k+1}\right)$ and its sharp bound given by $\left(X_{1};\ldots ;\hat{X_{i}};\ldots ;X_{k+1}\right).H\left(X_{i}\right)\leq H\left(X_{i}\right)$ is achieved in the case where $X_{k+1}$ is independent of $X_{1},\ldots ,X_{k}$ (we have $H_{k+1}=H_{k}+H\left(X_{k+1}\right)$ (higher dashed red line in Figure 4a). Hence, any entropy path lies in the (convex) entropy cone defined by the three points labeled $H_{k}$, $\min H_{k+1}$ and $\max H_{k+1}$: the three vertices of the cone depicted as a red surface in Figure 4a and called the Shannonian cone following Yeung’s seminal work [132]. The behavior of a mutual-information path and the bounds of its slope are richer and more complex than the preceding conditional entropy:

- For k=2, the conditional information is the conditional entropy $X_{i}.I\left(X_{j}\right)=X_{i}.H\left(X_{j}\right)$ and has the same usual bounds $0\leq X_{i}.I\left(X_{j}\right)\leq I\left(X_{j}\right)$.
- For k=3 the conditional mutual information $X_{i}.I\left(X_{j};X_{h}\right)$ is always positive or null $X_{i}.I\left(X_{j};X_{h}\right)\geq 0$ and hence $I_{2}\geq I_{3}$ ([92], p. 26, the opposite of Theorem 2.40, p. 30), whereas the higher limit is given by $X_{i}.I\left(X_{j};X_{h}\right)\geq \min\left(X_{i}.H\left(X_{j}\right),X_{i}.H\left(X_{h}\right)\right)$ ([34] th. 2.17), with equality iff $X_{j}$ and $X_{h}$ are conditionally independent given $X_{i}$ and implying that the slope from k=2 to k=3 increases in the $I_{k}$ landscape.
- For k>3, $X_{k}.I\left(X_{1};\ldots ;X_{k-1}\right)$ can be negative as a consequence of the preceding inequalities. In terms of information landscape this negativity means that the slope is positive, hence that the information path has crossed a critical point—a minimum. As expressed by Theorem due to Matsuda [34], $X_{k}.I\left(X_{1};\ldots ;X_{k-1}\right)<0$ iff $I_{k}<I_{k+1}$. The minima correspond to zeros of conditional information (conditional independence) and hence detect cocycles in the data. The results on information inequalities define as “Shannonian” [133,134,135] the set of inequalities that are obtained from conditional information positivity ($X_{i}.I\left(X_{j};X_{h}\right)\geq 0$) by linear combination, which forms a convex “positive” cone after closure. “Non-Shannonian” inequalities could also be exhibited [133,134], hence defining a new convex cone that includes and is strictly larger than the Shannonian set. Following Yeung’s nomenclature and to underline the relation with his work, we call the positive conditional mutual-information cone (the surface colored in red in Figure 4b) the “Shannonian” cone and the negative conditional mutual-information cone (the surface colored in blue in Figure 4b) the “non-Shannonian” cone.

#### 3.4.3. Information Paths Are Random Processes: Topological Second Law of Thermodynamics and Entropy Rate

Here we present the dynamical aspects of information structures. Information paths directly provide the standard definition of a stochastic process and it imposes how the time arrow appears in the homological framework, how time series can be analyzed, how entropy rates can be defined and so forth.

**Definition** **5.** *Random (stochastic) process ([136]): A random process $\left\{X_{t},t\in T\right\}$ is a collection of random variables on the same probability space (Ω,B,P) and the index set T is a totally ordered set.*

A stochastic process is a collection of random variables indexed by time—the probabilistic version of a time series. We have the following lemma:

**Lemma** **1.** *(Stochastic process and information paths): Let $\left(\Omega ,\Delta^{k},P\right)$ be a simplicial information structure, then the set of entropy paths ${\mathrm{HP}}_{k}$ and of mutual-information paths ${\mathrm{IP}}_{k}$ are in one-to-one correspondence with the set of stochastic processes $\left\{X_{t},t\in T,|T|=k\right\}$.*

**Proof.**  Considering each symbol of a time series as a random variable, the definition of a stochastic process corresponds to the unique information paths $HP_{i}$ and $IP_{i}$, whose total order is the time order of the series. More formally, we can prove that the number of different total order on the finite set T=1,2,…,k with *k* elements is k!, such that we can establish a one-to-one correspondence of total orders on *T* with permutations on *T* and by Theorem 2 with entropy and information paths. Let *T* be a finite set with *k* elements and ≤ a total order relation on *T*. Consider that k=1, then the set *T* contains only 1 element and any relation is trivially a total order on *T*. Now, consider that k>1 and that $T=\{t_{1},t_{2},\ldots ,t_{n}\}$. Suppose that x∈T. By the definition of a total order (Definition 9, p. 146 Bourbaki [137]), since ≤ is a total order on *T* then for *y* in *T* and where x≠y we have that x<y or y<x. If x<y,∀y∈T then we define *x* to be the minimum element in *T*. If x≠y,∀y∈T then there exists a y∈T such that y<x and such a process can be achieved recurrently until we obtain this minimal element. Without loss of generality, consider that $t_{1}$ is this minimal element. We then take the set $T\setminus \{t_{1}\}$ and repeat the preceding reasoning to find a minimal element $t_{2}$ of $T\setminus \{t_{1}\}$ and by recurrence we obtain $t_{1}<t_{2}<\ldots <t_{k}$ and hence that there are k! total orders in *T*. □

In other words, these paths are the automorphisms of {1,2…k}=[k]. We immediately obtain a topological version of the second law of thermodynamics, which follows from an elementary convexity property and improves the result of Cover [36]:

**Theorem** **3.** *(Stochastic process and information paths): Let $\left(\Omega ,\Delta^{k},P\right)$ be a simplicial information structure and let $X_{T}=\left\{X_{t},t\in T\right\}$ be a stochastic process defined by a collection of random variables on the same probability space (Ω,B,P) with cardinality $|X_{T}|=t$, where the index set T is a totally ordered set, then the entropy $H(X_{T};P)$, where $X_{T}$ is the joint-random variable of the variables in $X_{T}$ and can only increase or stay constant with t.*

**Proof.**  given the correspondence we just established in Lemma 1, the statement is equivalent to $H\left(X_{1},\ldots ,X_{k}\right)\geq H\left(X_{1},\ldots ,X_{k-1}\right)$ or $H\left(X_{1},\ldots ,X_{k}\right)-H\left(X_{1},\ldots ,X_{k-1}\right)\geq 0$ which by the chain rule of information (12) with k=−1/ln2 gives $\left(X_{1},\ldots ,X_{k-1}\right).H\left(X_{k}\right)\geq 0$. It is hence sufficient to prove the non-negativity of conditional entropy which can be found in Yeung ([92], p. 27). Consider the definition of conditional entropy given in 8, that we denote for simplicity:

(40) $$H\left(X_{k}|\left(X_{1},\ldots ,X_{k-1}\right);P\right)=k\sum_{\left(x_{1},\ldots ,x_{k-1}\right)}^{N_{1}\times \ldots \times N_{k-1}}p\left(x_{1},\ldots ,x_{k-1}\right).H\left(X_{k}|\left(\left(X_{1},\ldots ,X_{k-1}\right)=\left(x_{1},\ldots ,x_{k-1}\right)\right)\right),$$

with $\left(x_{1},\ldots ,x_{k-1}\right)\in \left(X_{1}\times \ldots \times X_{k-1}\right)$. It is hence sufficient to show that $H(X_{k}|\left(\left(X_{1},\ldots ,X_{k-1}\right)=\left(x_{1},\ldots ,x_{k-1}\right)\right))\geq 0)$, which follows from the fact that for $0\leq p_{\left(x_{1},\ldots ,x_{k-1}\right)}\left(x_{k}\right)\leq 1$, we have $k\ln(1/p_{\left(x_{1},\ldots ,x_{k-1}\right)}\left(x_{k}\right))\geq 0$ with k=−1/ln2, which follows from the concavity of the logarithm. The generalization with respect to the stationary Markov condition on $X_{T}$ used by Cover comes from the remark that in any case the indexing set of the variable is a total order. □

**Figure 5:**
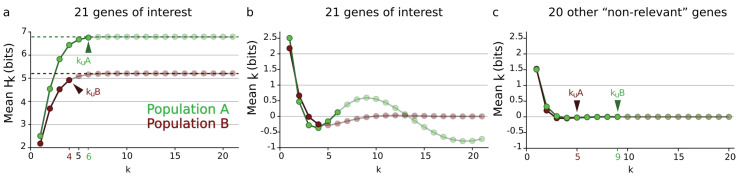
Example of mean entropy and information paths of gene expression. (**a**) Mean entropy path $\langle H_{k}\rangle$ for the 21 genes of interest for population A (green line) and population B neurons (red line). (**b**) Mean information path $\langle I_{k}\rangle$ for the same pool of genes. (**c**) Mean information path $\langle I_{k}\rangle$ for the remaining 20 genes (“non-relevant”). The undersampling dimension introduced in the associated paper [2] is depicted with arrows.

**Remark** **1.** *The equality $H\left(X_{1},\ldots ,X_{k}\right)=H\left(X_{1},\ldots ,X_{k-1}\right)$ corresponds to a statistical independence condition $I_{2}\left(\left(X_{1},\ldots ,X_{k-1}\right);X_{k}\right)=0$ and to an equilibrium condition. Note that the homological formalism imposes an “initial” minimally low entropy state H(0)=I(0)=0, which is a usual assumption in physics and which corresponds to the constant and zero degree homology, which has to have at least one component to talk about the cohomology. The meaning of this theorem in common terms was summarized by Gabor and Brillouin: “you cannot have something for nothing, not even an observation” [138]. This increase in entropy is illustrated in Figure 5a. The usual stochastic approach of time series assumes a Markov chain structure, imposing peculiar statistical dependences that restrict memory effects (cf. associated paper [2] proposition 8). The consideration of stochastic processes without restriction allows any kind of dependences and arbitrary long, historical and “non-trivial” memory. From the biological point of view, it formalizes the phenomenon of arbitrary long-lasting memory. From the physical point of view, without proof, such a framework appears as a classical analog of the consistent or decoherent histories developed notably by Griffiths [139], Omnes [140] and Gell-Mann and Hartle [141]. The information structures impose a stronger constraint of a totally ordered set (or more generally a weak ordering) than the preorder imposed by Lieb and Yngvason [142] to derive the second law.*

It is also interesting to note that even in this classical probability framework, the entropy cone (the topological cone depicted in Figure 4a) imposed by information inequalities, when considered with this time ordering, is a time-like cone (much like the special relativity cone) but with the arguably remarkable fact that we did not introduce any metric.

The stochastic process definition allows definition of the finite and asymptotic information rate:

**Definition** **6.** *Information rate: the finite information rate r of an information path $HP_{i}$ is $r=\frac{H_{k}}{k}$.*

The asymptotic information rate *r* of an information path $HP_{i}$ is $r=\lim_{k\to \infty }\frac{H_{k}}{k}$. It requires the generalization of the present formalism to the infinite dimensional setting or infinite information structures, which is not trivial and will be investigated in further work. We also let the question of the expression in information cohomology of the first principle as an open problem. Question: recently Baez and Fong published a Noether Theorem for Markov processes [39]; can we derive a Noether theorem for random discrete processes in general, that is, for all the symmetric groups $S_{n}$ using the present construction? Such a theorem would provide the topological expression of the first law of thermodynamics. This question was asked by Neuenschwander [40] and related to this aim, Mansfield gave a Noether theorem for finite elements [38].

#### 3.4.4. Local Minima and Critical Dimension

The derivative of information paths allows establishment of the lemma on which information path analysis is based. A critical point is said to be non-trivial if at this point the sign of the derivative of the path (i.e., the conditional information) changes.

**Lemma** **2.** *Local minima of information paths: if $X_{k}.I\left(X_{1};\ldots ;X_{k-1}\right)<0$, then all paths from 0 to $I_{k}$ passing by $I_{k-1}$ have at least one local minimum. In order for an information path to have a non-trivial critical point, it is necessary that k>3, the smallest possible dimension of a critical point being k=3.*

**Proof.**  it is a direct consequence of the definitions of paths and of conditional 2-mutual information $X_{k}.I_{2}$ positivity ($X_{k}.I_{2}\geq 0$, cf. Theorem 3.4.2.2 [92]). □

Note that, by definition, a local minimum can be a global minimum. If it exists, we will call the dimension *k* of the first local minimum of an information path the first informational critical dimension of the information path $IP_{i}$ and denote it $k_{i_{1}}$. This allows us to define maximal information paths:

**Definition** **7.** *Positive information path: A positive information path is an information path from 0 to a given $I_{k}$ corresponding to a given k-tuple of variables such that $I_{k}<I_{k-1}<\ldots <I_{1}$.*

**Definition** **8.** *Maximal positive information path: A maximal positive information path is a positive information path of maximal length. More formally, a maximal positive information path is a positive information path that is not a proper subset of positive information paths.*

The definitions make positive information paths and maximal positive information paths coincide with chains (faces) and maximal chains (facets), respectively. The maximal positive information path stops at the first local minimum of an information path, if it exists. The first informational critical dimension $k_{i_{1}}$ of a time series $IP_{i}$, whenever it exists, gives a quantification of the duration of the memory of the system.

#### 3.4.5. Sum over Paths and Mean Information Path

As previously, for k=1, $IP_{i}\left(1\right)$ can be identified with the self-internal energy and for k≥2, $IP_{i}\left(k\right)$ corresponds to the *k*-free-energy of a single path $IP_{i}$. The chain rule of mutual information (Equation (15)) and the derivative of an $IP_{i}$ path (Equation (38)) imply that the *k*-free-energy can be obtained from a single path:

(41) $$I_{k}=I\left(X_{1};\ldots ;X_{k};P\right)=I\left(X_{1}\right)-\sum_{i=2}^{k}X_{i}.I\left(X_{1};\ldots ;X_{i-1}\right)=IP_{i}\left(1\right)+\sum_{j=2}^{k}\frac{dIP_{i}\left(j\right)}{dj}.$$

Hence, the global thermodynamical relation (25) can be understood as the sum over all paths, the sum over informational histories: the classical, discrete and informational version of the path integrals in statistical physics [143]. Indeed, considering an inverse relation between time and dimension $t=\frac{1}{n}$ in the probability expression (32) for iid processes gives the usual expression of a unitary evolution operator $p_{••\ldots k\ldots •}=e^{\frac{t.E_{ij\ldots n}}{k}}$. Free-information-energy integrates over the simplicial structure of the whole lattice of partitions over degrees k≥2, which further justifies its free-energy name.

In order to obtain a single state function instead of a group of k! path functions, we can compute the mean behavior of the information structure, which is achieved by defining the mean $H_{k}$ and $I_{k}$, denoted $\langle H_{k}\rangle$ and $\langle I_{k}\rangle$:

(42) 〈Hk〉=∑T⊂[n];card(T)=kHk(XT;P)nk,

and

(43) 〈Ik〉=∑T⊂[n];card(T)=kIk(XT;P)nk.

For example, considering n=3, then $\langle I_{2}\rangle =\frac{I\left(X_{1};X_{2}\right)+I\left(X_{1};X_{3}\right)+I\left(X_{2};X_{3}\right)}{3}$. This defines the mean mutual-information path and a mean entropy path denoted 〈HP〉(k) and 〈IP〉(k) in the information landscape. The case k=2 of those functions introduced in Reference [108] is studied in Merkh and Montúfar [144] with a characterization of the degeneracy of their maxima and are called factorized mutual information. As previously, 〈IP〉(1) can be identified with the mean self-internal energy $U(X_{hom}^{n};P_{N})$ and for k>1〈IP〉(k) to the mean *k*-free-information-energy $G(X_{hom}^{n};P_{N})$, giving the usual isotherm relation:

(44) $$H\left(X_{hom}^{n};P_{N}\right)=U\left(X_{hom}^{n};P_{N}\right)-G\left(X_{hom}^{n};P_{N}\right).$$

The computation of the mean paths corresponds to an idealized information structure $X_{hom}^{n}$ for which all the variables would be identically distributed, would have the same entropy and would share the same mutual information $I_{k}$ at each dimension *k*: a homogeneous information structure, with homogeneous high-dimension *k*-body interactions. As is usually achieved in physics notably in mean-field theory (e.g., Weiss [145] or Hartree), it aims to provide a single function summarizing the average behavior of the system (we will see that in practice it misses the important biological structures, pointing out the constitutive heterogeneity of biological systems; see Section 4.1.3). Using the same dataset and results presented in References [2,3,108], the 〈IP〉(k) paths estimated on a genetic expression data set are shown for two populations of neurons (A and B) in Figure 5. We quantified the gene expression levels for 41 genes in two populations of cells (A or B) as presented in References [2,3,108]. We estimated $H_{k}$ and $I_{k}$ landscapes for these two populations and for two sets of genes (“genes of interest” and “non-relevant”) according to the computational and estimation methods presented in References [2,3,108]. The available computational power restricted the analysis to a maximum of n=21 variables (or 21 dimensions) and imposed us to divide the genes between the two classes “genes of interest” and “non-relevant”. The 21 genes of interest were selected within the 41 quantified genes according to their known specific involvement in the function of population A cells.

Figure 5 exhibits the critical phenomenon usually encountered in condensed matter physics, like the example of van der Waals interactions [146]. Like any $I_{k}$ path, 〈IP〉(k) can have a first minimum with a critical dimension $k_{i_{1}}$ that could be called the homogeneous critical dimension. For the 21 genes of interest (whose expression levels, given the literature, are expected to be linked in these cell types) the $\langle I_{k}\rangle$ path exhibited a clear minimum at the critical dimension $k_{i_{1}}=4$ for population A neurons and $k_{i_{1}}=5$ population B neurons, reproducing the usual free-energy potential in the condensed phase for which *n*-body interactions are non-negligible. For the 20 other genes, less expected to be related in these cell types, the $\langle I_{k}\rangle$ path exhibited a monotonic decrease without a non-trivial minimum, which corresponds to the usual free-energy potential in the uncondensed-disordered phase for which the *n*-body interactions are negligible. Indeed, as shown in the work of Xie and colleagues [147], the tensor network renormalization approach of *n*-body interacting quantum systems gives rise to an expression of the free-energy as a function of the dimension of the interactions, in the same way achieved here.

#### 3.4.6. Minimum Free Energy Complex

The analysis of information paths that we now propose aims to determine all the first critical points of information paths, in other words, to determine all the information paths for which conditional information stays positive and all first local minima of the information landscape that can also be interpreted as a conditional independence criterion. Such an exhaustive characterization would give a good description of the landscape and of the complexity of the measured system. The qualitative reason for considering only the first extrema for the data analysis is that beyond that point, mutual information diverges (as explained in Section 3.4.4) and the maximal positive information paths correspond to stable functional modules in the application to data (gene expression).

A more mathematical justification is that they define the facets of a complex in our simplicial structure, which we will call the minimum energy complex of our information structure, underlining that this complex is the formalization of the minimum free-energy principle in a degenerate case.

We now obtain the theorem that our information path analysis aims to characterize empirically:

**Theorem** **4.** ***(Minimum free energy complex):*** *the set of all maximal positive information paths forms a simplicial complex that we call the minimum free energy complex. Moreover, the dimension-degree of the minimum free energy complex is the maximum of all the first informational critical dimensions ($d=\max k_{i_{1}}$), if it exists, or the dimension of the whole simplicial structure n. The minimum free energy complex is denoted $X^{+d}$. A necessary condition for this complex not to be a simplex is that its dimension is greater than or equal to four (d≥4).*

**Proof.**  It is known that there is a one-to-one correspondence between simplicial complexes and their set of maximal chains (facets) (see Reference [148], p. 95, for an example). The last part follows from Lemma 2. □

**Figure 6:**
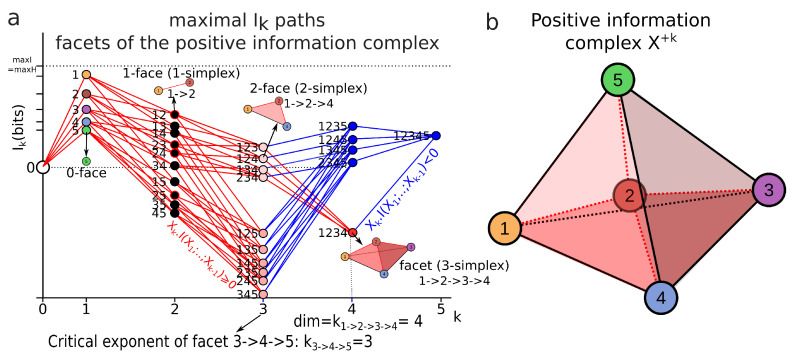
Example of maximal $I_{k}$ paths in an $I_{k}$ landscape for n=5 together with its corresponding minimum free energy complex. (**a**) Maximal $I_{k}$ paths in an $I_{k}$ landscape for n=5. The maximum positive information paths are depicted in red, for example, the paths 1→2→3→4 but also 4→3→2→1, 3→4→5 and 1→2→5 are maximum positive information paths (i.e., facets/maximal chains). The facet 1→2→3→4 is a 3-simplex while 3→4→5 is a 2-simplex with critical dimension $k_{3\to 4\to 5}=3$. The usual dimension of the simplex is used here but we could have augmented it by one, since we added the constant element “0” to the algebra (pointed space), such that the usual simplicial dimension and the critical dimension correspond. The maximal critical dimension of the positive information paths is the dimension of the complex and hence $d\left(X^{+k}\right)=d\left(1\to 2\to 3\to 4\right)=4$. (**b**) The minimum free energy complex corresponding to the preceding maximal $i_{k}$ paths. It is a subcomplex of the 4-simplex, also called the 5-cell, with only one four-dimensional cell among the five depicted as the bottom tetrahedron {1234} with darker red volume. It has 5 vertices, 10 edges, 10 2-faces and 1 3-face (cell), hence its Euler characteristic is $\chi (X^{+k})=5-10+10-1=4$ and its minimum free energy characteristic characteristic is: $H^{+k}\left(X^{+k}\right)=\sum_{X_{i}\in X^{+k}}^{5}I\left(X_{i}\right)-\sum_{\left(X_{i};X_{j}\right)\in X^{+k}}^{10}I\left(X_{i};X_{j}\right)+\sum_{\left(X_{i};X_{j};X_{h}\right)\in X^{+k}}^{10}I\left(X_{i};X_{j};X_{h}\right)-I\left(X_{1};X_{2};X_{3};X4\right)$.

In simple words, the maximal faces (e.g., the maximal positive information paths) encode all the structures of the minimum free energy complex. Figure 6 illustrates one of the simplest examples of a minimum free energy complex that is not a simplex, of dimension four in a five-dimensional simplicial structure of information $\Delta_{5}$.

We define the minimum free energy characteristic as:

(45) $$H^{+k}\left(X^{+k};P\right)=\sum_{i=1}^{k}{\left(-1\right)}^{i-1}\sum_{I\subset X^{+};card\left(I\right)=i}I_{i}\left(X_{I};P\right),$$

where the component with dimension higher than one is a free energy. In the example of Figure 6, it gives:

(46) $$\begin{array}{cc} H^{+k}\left(X^{+k}\right) & =\sum_{X_{i}\in X^{+k}}^{5}I\left(X_{i}\right)-\sum_{\left(X_{i};X_{j}\right)\in X^{+k}}^{10}I\left(X_{i};X_{j}\right)\\ & +\sum_{\left(X_{i};X_{j};X_{h}\right)\in X^{+k}}^{10}I\left(X_{i};X_{j};X_{h}\right)-I\left(X_{1};X_{2};X_{3};X4\right). \end{array}$$

We propose that this complex defines a complex system:

**Definition** **9.** *Complex system: A complex system is a minimum free energy complex.*

It has the merit to provide a formal definition of complex systems as simple as the definition of an abstract simplicial complex can be and to be quite consensual with respect to some of the approaches in this domain, as reviewed by Newman [149]. Notably, it provides a formal basis to define some of the important concepts in complex systems: emergence being the coboundary map, imergence the boundary map, synergy being information negativity, organization scales being the ranks of random-variable lattices, a collective interaction being a local minimum of free-energy, diversity being the multiplicity of these minima quantified by the number of facets, a network being a 1-complex, a network of networks being a 1-complex in hyper-cohomology.

The interpretation in terms of sum over paths in the complex is direct, as it sums over paths until conditional independence. We called it the minimum free energy complex but could instead have called it the positive or instantaneous complex because its facets appear as the boundaries of the “present” structure but it obviously contains all the past history and the memory of the structure (notably encoded in the negative $I_{k}$ that are necessarily non-Markovian). The topological formalization of the minimum energy allows the coexistence of numerous local minima—a situation usually encountered in complex systems (slow aging) such as frustrated glasses and K-sat problems [4,150] which settings correspond here to the case of *n* binary random variables, $N_{1}=\ldots =N_{2}=2$. The existence of the frustration effect, due to the multiplicity of these local minima in the free energy landscape [151], has also been one of the main difficulties of condensed matter theory. Matsuda could show that $I_{k}$ negativity is a signature of frustration [34]. The first axioms of DFT consider that probability densities of $n^{\prime }$ elementary bodies are each in a 3-dimensional space [117,118], defining a whole simplicial structure of dimension $n=3n^{\prime }$, commonly called the configuration space. When considered with the physical axiom of a configuration space, Theorem 4 implies that, while the minimum free energy complex of an elementary body can only be a simplex, the configuration space of $n^{\prime }$ elementary bodies can be a complex with (quite) arbitrary topology. In simple terms, this settles the elementary components of the configuration space as 3-simplices, whose composition can give arbitrarily complicated *k*-complexes. This idea is in resonance with the triangulations of space-time that arose notably from the work of Wheeler [119] and Penrose [12], like spin foams [152] and causal sets [153], while here we only considered classical probabilities.

## 4. Discussion

### 4.1. Statistical Physics

#### 4.1.1. Statistical Physics without Statistical Limit? Complexity through Finite Dimensional Non-Extensivity

The measure of entropy and information rate on data (the evolution of entropy $H_{k}$ when the number of variables *k* increases) has a long history. Originally, in the work of Strong and colleagues [154] and as usual in information theory and statistical physics, it was considered that the “true” entropy was given in the asymptotic limit $\lim_{n\to \infty }H_{n}$ under stationarity or stronger assumptions. As explained in Section 2.1 (see also the note of Kontsevitch [76], in the work of Baez, Fritz and Leinster [81]) and extensively in the statistical physics works of Niven [155,156,157], entropy does not need asymptotic or infinite assumptions such as the Stirling approximation to be derived. Rather, entropy appears without approximations in a discrete formalism equivalent to Galois cohomology [103] as a first cohomology class which has to be completed by invariants in the higher degrees. Here and in the associated paper [2], we have tried to understand, explore and exploit this observation. Rather than being interested in the asymptotic limit (the infinite dimensional case) and absolute values of information, the present analysis focuses on the finite version of the “slow approach of the entropy to its extensive asymptotic limit” that Grassberger [158] as well as Bialek, Nemenman and Tishby proposed to be “a sign of complexity” [159], “complexity through non-extensivity” (see also Tsallis [160]). In short, we consider the non-extensivity of information before considering its asymptotic limit. Considering a statistical physics without statistical limit could be pertinent for the study of “small” systems, which concerns biological systems. Their small size allows them to harness thermal fluctuations and impose their investigation with out-of equilibrium methods, as exposed in the work of Ritort and colleagues, reviewed in Reference [161]. Finiteness and discreteness could be an essential property of the physics of complex living systems. The $H_{k}$ and $I_{k}$ landscapes presented here give a detailed expression of the “signs of complexity” and non-extensivity for such small size systems (finite dimension *k*) and give a finite dimensional geometric view of the “slow approach of the entropy to its extensive asymptotic limit”. In a sense, what replaces here the large number limits, Avogadro number consideration and so forth, is the combinatorial explosions of the different possible interactions: in the same way as in van der Waals paradigm, a combinatorial number of weak interactions can lead to a strong global interaction. Among all possible data structures, one is universal: data and empirical measures are discrete and finite, as emphasized by Born [16] and fully justify the cohomological approach used here originating in topos (designed by Grothendieck to hold the discrete and continuous in a single hand [162]), which was originally constructed to handle the Lie and Galois theory, as well as continuous and discrete symmetries, in a common framework. Notably, the principle originally enunciated by Willard Gibbs which considers that a phase transition is a singularity in thermodynamic behavior and occurs only in infinite systems is physically false: none of the observed systems are infinite and many of them—even small—present phase transition [35]. Cohomological methods allow the Gibbs transition principle to be corrected with respect to experience while leaving the thermodynamic theory untouched.

#### 4.1.2. Naive Estimations Let the Data Speak

One of the striking results of the data analysis as presented here and in the associated paper [2] concerns the relatively low sample size (m=41 and m=111 for the analysis with cells as variables and with genes as variables respectively) required to obtain satisfying results in relatively high dimensions (k=10 and k=6, respectively). Satisfying results means here that they predict already-known results reported in the biological literature, or in agreement with experts’ labels. In Reference [97], Nemenman and colleagues, who developed the problematic of the sampling problem, state in the introduction that “entropy may be estimated reliably even when inferences about details of the underlying probability distribution are impossible. Thus the direct estimation of dependencies has a chance even for undersampled problems” and conclude that “a major advantage of our definition of statistical dependencies in terms of the MaxEnt approximations is that it can be applied even when the underlying distributions are undersampled”. The present analysis agrees and confirms their conclusion. The methods applied here are quite elementary. They do not make assumptions of an expected or true distribution, of maximum entropy distribution or pairwise interaction Hamiltonian, coupling constant or metric, of stationarity or ergodicity or i.i.d. process, Markov chain, or underlying network structure, or whatever prior that would speak in place of the data. It just considers numerical empirical probabilities as expressed by Kolmogorov axioms ([163], chap. 1), which he called the “generalized fields of probability” because they do not assume the sixth axiom of continuity. Rather than fixing a model with priors, the present formalism allows the raw data to freely impose their specific structure to the model, which is usually called the naive approach or naive estimation. If one accepts that a frequentist theory and interpretation of probability is mathematically valid ([163], chap. 1), one may then conclude that a frequentist theory of entropy and information may also hold and moreover directly fulfills the usual requirement of observability in theoretical physics recalled by Born in his Nobel lecture [16]. This frequentist elementary consideration is not mathematically trivial, notably when considered from the number-theoretic point of view. For example, the combinatoric of integer partitions of *m* could be investigated in the general information structure (partition) context, which to our knowledge has not been achieved in the context of probability and information.

#### 4.1.3. Discrete Informational Analog of Renormalization Methods: No Mean-Field Assumptions Let the Objects Differentiate

To our best knowledge, all previous studies that tried to quantify statistical dependences using information methods with more than three variables used total correlation [97,164] and crucially assumed that the interaction between the variables are homogeneous, which corresponds to the usual mean field assumption and to the identically distributed case of mean information (Section 3.4.5) presented here. The proposed combinatorial decomposition allows heterogeneous classes within the set of variables to be identified [2,3], which would not have been possible using homogeneous assumptions, just as renormalization methods allowed the failures of mean field models to be overcome [41].

#### 4.1.4. Combinatorial, Infinite, Continuous and Quantum Generalizations

In place of the usual assumptions of statistical physics and in order to compute effectively information structures on the data, we had to concede a severe restriction to the simplest simplicial combinatoric, leaving the whole multinomial combinatoric as potential and relevant extensions of the present data analysis. It is likely (and left as an open question) that the generalization of the binomial combinatoric presented here to the multinomial case will allow extension of the theorem obtained in the associated paper that shows that informations provide co-ordinates on the probability simplex for binary variables (Theorem 3 in Reference [2]). Even larger extensions have already been achieved theoretically, notably by Vigneaux, who developed *q*-multinomial deformations of this combinatorics associated with Tsallis entropies [19,165,166]. Moreover, as shown in the PhD of Vigneaux, this finite and discrete elementary setting extends nicely to the infinite and continuous case [19] within cohomology theory. If those structures currently appear gigantic and out of computational reach, one should expect that the future generations of computing devices will allow us to investigate them. On the side of the generalization of the information cohomology to quantum information [1,19] (see also Maniero [21]), Adami and Cerf showed that quantum conditional entropies and $I_{2}$ can be negative and that it happens precisely for entangled systems according to Bell inequalities [167,168]. The framework of Adami and Cerf provides a natural way to generalize the present classical $I_{k}$ structures to the quantum case and allows the interpretation that classical $I_{k}$ negativity detects classical entanglement-like relations. According to their results, Bell inequalities impose that the “non-Shannonian” (quantum) cone in information landscapes should happen below the third dimension: in contrast to what we presented for the classical minimum energy complex in Section 3.4.6, quantum entanglement should allow non-simplex quantum minimum free energy complexes to happen in dimension below 3—a promising topological insight into the possible non-locality of the configuration space.

### 4.2. Data Science

#### 4.2.1. Topological Data Analysis

Thanks to the recent success of topological data analysis, a recurrent question has concerned the relation of information cohomology with persistence homology. The answer is not easy, since both appeared independently and are quite different in formalism, algorithms and results on the dataset. It is possible to provide an intuitive non-rigorous interpretation of the present work in persistence terms. Most of the persistent methods consist of approximating the birth and death of the Betti’s numbers of the $\overset{˘}{C}$ech complex obtained by considering balls around each data point while the radius of the ball grows. The $\overset{˘}{C}$ech complex is given by the intersection of the balls and for combinatorial computational reasons, most of the algorithms restrict to pairwise intersections, giving the Vietoris–Ripps complex as an approximation of the $\overset{˘}{C}$ech complex. Our method focuses on the intersection of random variables rather than balls around data points: a theorem of Hu Kuo Ting [91] (Theorem 1 in Reference [2]) shows the equivalence of mutual-information functions with set theoretic finite measurable functions endowed with the intersection operator, formalizing the usual naive Venn diagram interpretation of mutual information. Hence, leaving the mathematical rigor to allow an interpretation of the current algorithm in the common language of persistence, I compute here an information-theoretic analog of the $\overset{˘}{C}$ech complex and it is not excluded that this analogy can be made formal and notably to establish some nerve theorem for information structures (see Oudot for a review [169]). It turns out that “zero information intersections” is exactly equivalent to statistical independence (Theorem 2 in Reference [2]). Then, the balls can be viewed as a local estimation of the probability density under a metric space assumption, while computing the persistence is roughly analog to varying the graining of the probability estimation as achieved in Section 5.6 of Reference [2] and the maximal mutual-information coefficient method proposed by Reshef et al. [116]. Hence, in regard to current topological data analysis methods, the methods presented here provide an intrinsically probabilistic cohomological framework: the differential operators are fundamental maps in probability-information theory. As a consequence, no metric assumption is required a priori: in practice, it is possible to compute the homology, for example, on position variables and/or on qualitative variables such as “nice” and “not nice” or “Alice” and “Bob”. The present method is topological and avoids the a priori introduction of such a metric; rather, a family of Shannon’s pseudometric emerges from the formalism as a first cohomological class (Section 2.3.1). Considering a symmetric action of conditioning, we obtain Shannon’s metric parametrized by a scalar multiplicative constant.

#### 4.2.2. Unsupervised and Supervised Deep Homological Learning

As underlined in the Introduction, complexes of random variables and minimum free energy complex can be understood as providing a geometrically constrained architecture to deep neural networks, where the depth of the neural network corresponds to the dimension of the cochain complex and marginal variables and information $I_{1}$ correspond to the input layer. Notably, the multiplicity of facets forming the complexes allows consideration of neural networks with parallel layer architecture to analyze conditionally independent features of the input, as effectively achieved in real nervous systems, such as the macroscopic “where and what” (dorso and ventral, respectively) visual processing streams in the vertebrate cortex [170,171]. Moreover, the energy functional interpretation of mutual information $I_{k}$ and of total correlation $G_{k}$ functions directly follow and generalize the definitions of the hierarchical deep architectures of Boltzmann machines and of Helmholtz machines [65,66] that explicitly expressed free energy in terms of Kullback-Leibler (KL) divergences between layers. Notably, the introduction of the multiplicity decomposition of “energy functions” formalizes unsupervised learning in neural networks in terms of a combinatorial family of analytically independent functions $I_{k}$ with independent gradients (Theorem 4 in Reference [2]): instead of a single energy and associated gradient descent, mutual information provides a multiplicity of gradients. The application scope is restricted to the general problem of unsupervised learning here but the supervised subcase can be proposed from this and is detailed with some examples of application to the digits MNIST images dataset presented here [63] and in a future publication. Notably, the mathematical depth of the back-propagation algorithm [172,173,174] comes from the fact that it implements the chain rule for derivation with respect to the parameters, allowing learning in the class of differentiable functions. In information cohomology, supervised learning appears as a subcase of unsupervised learning and defines the sublattice, information landscapes and complexes for which all chains contain the label variable $X_{i}$ to be learned and the $2^{n-1}$$H_{k}$, $I_{k}$ which (sub)gradients are independent in open dense subsets of the probability subsimplex $\Delta_{X/X_{i}}$ (subsimplex obtained by conditioning on the parameters $E_{X_{i}}$, the conditional probability laws with respect to $X_{i}$). Notably, the marginal degree 1 layer is only composed of the label variable $X_{i}$, that correponds to the usual definition of an input, and the simplicial structure is translated by one degree. Then, the maximal depth of a deep neural network achieving the classification given the data is the dimension of the information simplicial complex—a result that can be linked to the work of Montúfar on the dimension of restricted Boltzmann machine [175]. In this cohomological context, the back-propagation is implemented by the information chain rule and is forward as imposed the cohomology contra-variant nature. The relation to information geometry and Amari’s natural gradient [176] follows from the relation of the Fisher information with the Hessian of the KL divergence [177] (or entropy [178]) and from the implementation of natural gradients for deep network training [179] and is left for further courageous investigations.

#### 4.2.3. Epigenetic Topological Learning—Biological Diversity

**Figure 7:**
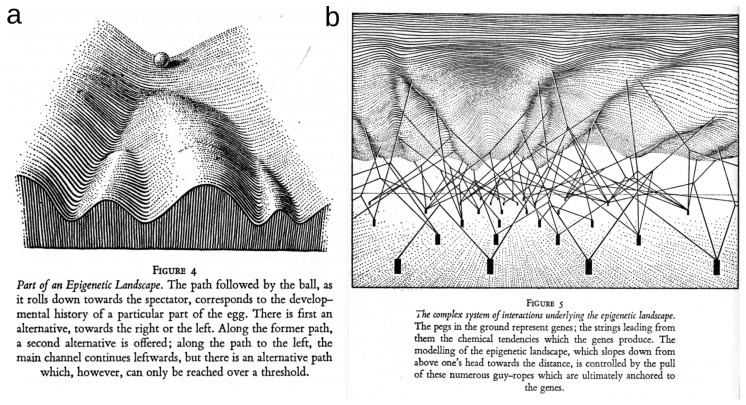
The epigenetic landscape of Waddington. (**a**) The epigenetic landscape of Waddington. A path of the ball in this landscape illustrates a cell’s developmental fate. (**b**) “The complex system of interactions underlying the epigenetic landscape”, with Waddington’s original legends [180].

In place of the MaxEnt principle, we proposed a least energy principle equivalent here to a homological complex (finite and without metric assumptions). Mathematically, profited from the fact that whether the maximum of entropy functional is always unique and in a sense normative, the minima of $I_{k}$ functionals exhibit a rich structure of degeneracy, corresponding to the “non-Shannonian set” [133,134,135] and conjectured to be at least as rich as topological links can be [2]. We proposed that this multiplicity of minima accounts for biological diversity, or more precisely that the number of facets of this complex quantifies the diversity in the system. The application to cell type identification presented in the associated paper [2] gives a preliminary validation of this quantification. Moreover, the definition of a complex system such as the minimum free energy complex given in Section 3.4.6, underlining that diversity is just the multiplicity of the minima, is in agreement with Waddington’s original work [180] (see Figure 7b). In the allegory of Waddington’s epigenetic landscapes, whatever the ball, it will always fall down—a statement that can be assimilated to the second law of thermodynamics. Doing so, however, it will be able to take different paths: diversity comes from the multiple minima. Waddington’s explanation of this landscape is as a “complex system of interactions” that can be formalized by the minimum free energy complex with interactions corresponding to the $I_{k}$. Moreover, formalisms assuming that the variables are identically distributed, as for the homogeneous systems described in the section on mean paths (Section 3.4.5), will display a single first minima (one facet, a simplex) and hence no diversity. Sharing the same aims, Teschendorff and Enver and then Jin and colleagues, proposed an alternative interpretation of Waddington’s landscape in terms of signaling entropy [181] and of probability transitions [182], respectively.

Following Thom’s topological morphogenetic view of Waddington’s work [183], we propose that $I_{k}$ landscape, paths and minimum free energy complex provide a possible informational formalization of Waddington’s epigenetic complex landscape and cell fates (cf. Figure 7). This formalization of Waddington’s epigenetic view is consistent with the machine learning formalization of Hebbian epigenetic plasticity. From the pure formal view, the models of Hebbian neural learning like Hopfield’s network, Boltzmann machines, the Infomax models proposed by Linsker, Nadal and Parga, as well as Bell and Sejnowski [184,185,186]) can be viewed as binary variable subcases of a generic *N*-ary variable epigenetic developmental process. For example, Potts models were implemented for the simulation of cell-based morphogenesis by Glazier and colleagues [187]. Hence, the topological approach can allow the treatment of neural learning and development on the ground of a common epigenetic formalism, in agreement with biological results pointing out the continuum and “entanglement” of the biological processes underlying development and learning [188]. In terms of the current problematics of neuroscience, such generalization allows, on a formal level, consideration of an analog coding in place of a digital coding and the methods developed here can be applied to studies investigating (discrete) analog coding.

Moreover, following all the work of these past decades on the application of statistical physics to biological systems (some of them cited in this article), we propose that the epigenetic process implements the first two laws of thermodynamics, weak topological versions of which are proposed to hold in the raw data space (without phase space or symplectic structure, cf. Section 3.4.3). As previously underlined, the condition for such an inscription of living organism dynamics into classical statistical physics to be legitimate is that the considered variables correspond to phase space variables.

## 5. Conclusions

In this paper we have proposed a unified view and account for classical statistical physics and machine learning methods like topological data analysis and deep neural networks under a common information cohomology framework. The particularity and main novelty of the methods are that they are finite and discrete and that such axioms, rather than being a default, can have fundamental mathematical (Galois theory [103]), probabilistic (Kolmogorov foundations of finite probability field [163]), physical (frequentist hypothesis and observability axiom [16]) and computational (directly computable combinatorial expressions) and machine learning (numerical pattern classification) meaning. From the statistical physics point of view, we developed within the information cohomology framework an expression of internal and free energy, of the second law and conjecturally of the first law of thermodynamics, while developing a generic *k*-body interaction model that can be viewed as a dicrete-finite and informational analog to renormalization methods (and hence more physically sound [44,45]), avoiding mean-field approximations. The application of these informational methods to genetic expression data reproduced the usual signatures of phase transition of mean-field *k*-body models [46,47,48]. From the machine learning point of view, this paper provides a combinatorial and computational expression of the information cohomology and notably of the simplicial subcase that can be effectively computed and underline its universal classifier function. The methods applied in References [2,3] offer new algorithms for topological data analysis which are intrinsically probabilistic, as well as acohomological insight into deep neural network architecture and training/learning algorithms, both supervised and unsupervised, while providing a generic biological model of epigenetic plasticity and development.

## Supplementary Materials

The software Infotopo is available at https://github.com/pierrebaudot/INFOTOPO.

## Abbreviations

The following abbreviations are used in this manuscript:

iid	Independent identically distributed
$H_{k}$	Multivariate *k*-joint Entropy
$I_{k}$	Multivariate *k*-mutual information
$G_{k}$	Multivariate *k*-total-correlation or *k*-multi-information
